# Identification of *WRKY* gene family and characterization of cold stress-responsive * WRKY* genes in eggplant

**DOI:** 10.7717/peerj.8777

**Published:** 2020-03-17

**Authors:** Yan Yang, Jun Liu, Xiaohui Zhou, Songyu Liu, Yong Zhuang

**Affiliations:** Jiangsu Key Laboratory for Horticultural Crop Genetic Improvement, Institute of Vegetable Crops, Jiangsu Academy of Agricultural Sciences, Nanjing, China

**Keywords:** Eggplant, WRKY transcription factor, Phylogenetic analysis, Inton-exon structure, Conserved motifs, Differentially expressed, Cold stress

## Abstract

**Background:**

WRKY proteins play a vital role in the plants response to different stresses, growth and development. Studies of WRKY proteins have been mainly focused on model plant Arabidopsis and a few other vegetable plants. However, the systematical study of eggplant WRKY transcription factor superfamily is scarce.

**Methods:**

Bioinformatics has been used to identify and characterize the eggplant *WRKY* gene family. For the exploration of the differentially expressed *WRKY* genes, two cultivars with different cold-tolerance were used. Finally, we performed a virus-induced gene silencing (VIGS) experiment to verify the functions of *SmWRKY26* and *SmWRKY32*.

**Results:**

Fifty eight (58) genes encoding eggplant WRKY proteins were identified through searching the eggplant genome. Eggplant WRKY proteins could be classified into three groups or seven subgroups in accordance with other plants. WRKY variants were identified from the eggplant. Gene structure analysis showed that the number of intron in eggplant WRKY family was from 0 to 11, with an average of 4.4. Conserved motif analysis suggested that WRKY DNA-binding domain was conserved in eggplant WRKY proteins. Furthermore, RNA-seq data showed that *WRKY* genes were differentially expressed in eggplant response to cold stress. By using VIGS, the two differentially expressed genes-*SmWRKY26* and *SmWRKY32* were verified in response to cold stress.

**Discussions:**

This study provides a foundation for further exploring the functions of WRKY proteins in eggplant response to stresses and eggplant genetic improvement in stresses.

## Introduction

WRKY proteins, a family of transcription factor specific in plants, have been identified for more than twenty years since they were first named as SPF1 in sweet potato (*Ipomoea batatas* L.) and seldom found in other species (Ishiguro & Nakamura, 1994; Eulgem et al., 2000; Rinerson et al., 2015). They are named as WRKY because of their highly conserved WRKY domain present in the protein sequence. The WRKY domain consists of a conserved core sequence WRKYGQK and a zinc-finger motif (Eulgem et al., 2000). WRKY proteins can be classified into three groups based on the number and amino acid sequences of the conserved WRKY domains. Group I WRKY proteins contain two WRKY domains, while the other two WRKY groups compose of one. The zinc finger motif is different among the three WRKY groups. A C2H2 motif is found in the C-terminal of group I and II WRKY proteins. Instead of C2H2 motif, a C2HC motif is in the C-terminal of group III WRKY proteins (Eulgem et al., 2000). Moreover, group II WRKY proteins can be further divided into five subgroups in accordance with the number and type of amino acids between the C2 in the zinc finger motif (Rushton et al., 2010). WRKY transcription factors can specially bind to the target genes by recognizing the (T)TGAC(C/T) sequences in the promoters of target genes (Yu, Chen & Chen, 2001).

Since its first identification in sweet potato, the functions of WRKY proteins have been focused on biological progresses, including plant responses to biotic stress, abiotic stress and plant development. Several studies have confirmed that WRKY transcription factors are involved in hair development (Verweij et al., 2016), seed size (Luo et al., 2005; Gu et al., 2017), pollen development (Lei et al., 2017), growth types (Yang et al., 2016), flowering time (Yang et al., 2016), fruit ripening (Cheng et al., 2016) and leaf senescence (Ay et al., 2009; Brusslan et al., 2012; Yang et al., 2016). WRKY transcription factors modulates plant hormone signaling pathways (Liu et al., 2017a; Liu et al., 2017b; Jin et al., 2018; Zheng et al., 2018). Despite the important roles in plant growth and development and signal pathways, the most significant function of WRKY proteins studied thoroughly and reported widely is transcription regulation of response to abiotic and biotic stresses. In *Arabidopsis thaliana*, AtABO3, a WRKY protein, was involved in plant response to drought stress (Ren et al., 2010). In rice (*Oryza sativa* L.). OsWRKY11 was induced by heat stress and overexpression of the *OsWRKY11* enhanced the tolerance to high temperature stress (Wu et al., 2009). In addition, a vast number of studies reported that WRKY transcription factors were correlated with the resistance to salt stress (Xu et al., 2014; Cai et al., 2017), ozone stress (Gottardini et al., 2016; Zhang et al., 2017) and cold stress (He et al., 2016; Zhang et al., 2016). Futhermore, the functions and roles of WRKY proteins in plant immune response are outstanding. For example, Xu et al. (2006) found that AtWRKY18, AtWRKY40 and AtWRKY60 interacted with each other physically and functionally in an overlapping and antagonistic pattern, which played distinct roles in plant response to two types of pathogens-*P.syringae* and *B.cinerea*. Molecular complementation and gene silencing have confirmed that WRKY33 homologues in Arabidopsis and tomato (*Solanum lycopersicum* L.) played a critical role in resistance to botrytis (Zhou et al., 2015). In a recent report, *BROWN PLANTHOPPER RESISTANCE 14* (*BPH14*), the first planthopper resistance gene, mediated the planthopper resistance by interaction with WRKY46 and WRKY72 that could bind to receptor-like cytoplasmic kinase genes and the callose synthase genes in rice. This result threw a light on the role of WRKYs in plantthopper resistance (Hu et al., 2018). Taken together, it is obvious that WRKY proteins can mediate plant defense mechanism in different manners.

Reports about WRKY proteins in some crops including vegetable crops are relatively limited, although all studies about WRKY proteins have focused mainly on model plant Arabidopsis and a few other plants. For instance, there are more than 70 WRKY proteins in Arabidopsis (Eulgem et al., 2000; Dong, Chen & Chen, 2003), 102 WRKY proteins in rice (Ross, Liu & She, 2007), 174 WRKY proteins in soybean (*Glycine max* L.) (Yang et al., 2017a; Yang et al., 2017b) and 81 WRKY proteins in tomato (Huang et al., 2012a; Huang et al., 2012b). Eggplant, a world-wide cultivated species, is one of the most important vegetable crops (Wang, Sulli & Fu, 2017; Yang et al., 2017a; Yang et al., 2017b). It is especially popular in Asia, the Middle and Near East, Southern Europe and Africa (Daunay & Lester, 1988). The information of eggplant genome has been released in 2014 (Hirakawa et al., 2014). Several gene families were identified, such as *CBL* and *CIPK* genes (Li et al., 2016), R2R3MYB transcription factor superfamily (Wang et al., 2016), calcium-dependent protein kinases (CDPKs) (Kumari et al., 2017) and *CBF* genes (Zhou et al., 2018a; Zhou et al., 2018b). Previously, Yang et al. (2015) have identified the WRKY proteins in *Solanum melongena* L. and *Solanum torvum* Sw. using the RNA-sequencing data, respectively. Herein, we identified 58 WRKY transcription factors in eggplant through genome-wide analysis. Subsequently, a comprehensive analysis of eggplant *WRKY* genes was carried out based on their sequences and structures, phylogenetic relationship and expression. Meanwhile, several *WRKY* genes were found to actively respond to cold stress. *SmWRKY26* and *SmWRKY32* were preliminarily verified involved in regulating eggplant tolerance to cold stress through VIGS. Therefore, cold-responsive genes in eggplant genome were preliminarily validated and the transcript level of these responsive genes may influence eggplant response to cold stress. The results threw light on their probable functions in eggplant response to abiotic stresses and provided a reference for genetic improvement in the stress tolerance.

## Materials & Methods

### Plant treatment

In the present study, the seedlings of ‘sanyueqie’ eggplant variety were germinated and grown in a growth chamber at 28 °C with a 16/8 h light/dark photoperiod. The cotyledons of plants were inoculated with the cultures at the cotyledon stage and transferred to 22 °C to induce gene-silencing. When the fourth leaves were fully expanded, the seedlings were transferred into 4 °C for five days, followed by three days of recovery at 28 °C.

### Identification of WRKY family in eggplant

To identify the WRKY proteins in eggplant, two methods were employed. We searched the eggplant genome database (http://eggplant.kazusa.or.jp/) for WRKY proteins based on the sequences of conserved WRKY domains in Arabidopsis (Dong, Chen & Chen, 2003). All peptide sequences of eggplant were download from the eggplant database. The Hidden Markov Model (HMM) of WRKY was used to BLASTP. The candidate WRKY transcription factors were obtained subsequently. The Pfam databases (http://pfam.xfam.org/) and SMART databases (http://smart.embl-heidelberg.de/) were used to validate the candidate WRKY proteins. ProtParam tool (https://web.expasy.org/protparam/) was used to predict the molecular weights (MWs) and isoelectric points (pIs) of WRKY proteins.

### Phylogenetic analysis of SmWRKY proteins

To understand the evolutionary relationship between the eggplant and other plants, multiple sequence alignment was constructed using CLUSTAL W based on the conserved WRKY domain sequences of WRKY proteins from eggplant, tomato, Arabidopsis and rice (Thompson, Higgins & Gibson, 1994; Ross, Liu & She, 2007). To know the relationship among eggplant WRKY proteins, the phylogenetic tree of eggplant WRKY proteins was conducted with the conserved WRKY domain sequences of WRKY proteins from eggplant. Phylogenetic analysis was performed with MEGA v5.1 (Tamura et al., 2011; Hall, 2013). A phylogenetic tree was produced following the neighbor-joining method.

### Intron-exon structure and conserved motifs analysis

The intron-exon structures of *SmWRKY* genes were determined by submitting a txt file of gene features with gff3 format to Gene Structure Displayer Server (http://gsds.cbi.pku.edu.cn/) (Hu et al., 2015) (Data S1). The conserved motifs in the WRKY proteins were predicted using the MutipleEm for Motif Elicitation (MEME) program (http://alternate.meme-suite.org/tools/meme). The parameters in this study were set as follows: 10 motifs should be found, minimum width was 15, maximum width was 50. Interpro Scan was used to annotate the ten motifs.

### Virus-induced gene silencing (VIGS)

Differentially expressed *WRKY*s were identified from the RNA-seq data conducted previously. *SmWRKY26* and *SmWRKY32* were up-regulated in both cultivars after 4 °C treatment and the proteins were encoded by two genes with a high sequence similarity. We selected these two genes to performed a functional verification through VIGS. The fragments of *SmWRKY26* and *SmWRKY32* were amplified using the specific primers (Data S2), verified using VIGS tool in SGN (http://vigs.solgenomics.net/) and cloned to TRV (tobacco rattle virus) RNA2 vector (Fu et al., 2005; Huang et al., 2012a; Huang et al., 2012b). The empty vector TRV RNA2 was used as the control. The pTRV1, pTRV2, pTRV2-*SmWRKY26* and pTRV2-*SmWRKY32* were introduced into *agrobacterium* GV3101 with electroporation method, respectively. The mixture of *Agrobacterium* cultures containing pTRV1 and pTRV2 (1:1, v/v), pTRV1 and pTRV2-*SmWRKY26* (1:1, v/v) and pTRV1 and pTRV2-*SmWRKY32* (1:1, v/v) at OD_600_ = 1 were incubated for 4 h in the dark at room temperature before inoculation. Three independent replicates were performed.

### Quantative real time-polymerase chain reaction (qRT-PCR)

Specific primers were designed in the website (http://primer3.ut.ee/) (Data S2) (Koressaar & Remm, 2007). Total RNA was isolated from the tissue samples using the Trizol reagent according to the manufacturer’s instruction. Subsequently, RNA was reverse transcribed using the HiScript II Q Select RT SuperMix for qPCR kit (Vazyme, China). qRT-PCR was performed on an LightCycler 480 II (Roche, Switzerland) using the AceQ®qPCRSYBR® Green Master Mix (Vazyme, China). The PCR conditions consisted of denaturation at 95 °C for 5 min, followed by 40 cycles of denaturation at 95 °C for 10 s, annealing and extension at 60 °C for 30 s. Melt curve analysis was performed to determine the specificity of reactions. The transcript level was calculated according to the ΔΔCt method (Livak & Schmittgen, 2001). The eggplant *Actin* gene (Sme2.5_01462.1) was used as internal control. Three biological and technical repetitions were performed during the whole experiment.

**Table 1:** The WRKY proteins in eggplant.

Protein name	Gene ID	Group	Amino acid	Molecular weight	pI
SmWRKY1	Sme2.5_00009.1_g00017.1	3	340	38.90	5.14
SmWRKY2	Sme2.5_00013.1_g00024.1	1	511	57.35	6.58
SmWRKY3	Sme2.5_00013.1_g00025.1	3	360	40.79	5.70
SmWRKY4	Sme2.5_00016.1_g00020.1/pseudo	2c	338	37.43	5.58
SmWRKY5	Sme2.5_00029.1_g00022.1	2d	324	35.87	9.57
SmWRKY6	Sme2.5_00038.1_g00016.1/TE	2d	367	40.39	9.72
SmWRKY7	Sme2.5_00100.1_g00012.1	1	595	64.89	6.56
SmWRKY8	Sme2.5_00161.1_g00022.1	1	490	54.66	5.71
SmWRKY9	Sme2.5_00196.1_g00004.1	1	989	109.90	6.58
SmWRKY10	Sme2.5_00232.1_g00014.1	2d	239	27.26	5.55
SmWRKY11	Sme2.5_00232.1_g00015.1	2d	285	30.87	5.53
SmWRKY12	Sme2.5_00264.1_g00017.1	2d	306	33.62	9.64
SmWRKY13	Sme2.5_00281.1_g00010.1	2b	454	50.55	5.92
SmWRKY14	Sme2.5_00386.1_g00007.1	2b	561	61.09	6.89
SmWRKY15	Sme2.5_00423.1_g00013.1	2e	277	30.16	5.51
SmWRKY16	Sme2.5_00556.1_g00018.1	2a	224	25.48	6.45
SmWRKY17	Sme2.5_00556.1_g00019.1	2a	261	29.75	5.75
SmWRKY18	Sme2.5_00574.1_g00007.1	2c	171	19.67	5.40
SmWRKY19	Sme2.5_01030.1_g00008.1	1	486	54.38	6.50
SmWRKY20	Sme2.5_01060.1_g00010.1	2d	402	45.04	9.43
SmWRKY21	Sme2.5_01071.1_g00003.1	2e	297	33.64	6.45
SmWRKY22	Sme2.5_01077.1_g00010.1	1	649	71.02	6.46
SmWRKY23	Sme2.5_01130.1_g00003.1	2b	334	37.60	7.01
SmWRKY24	Sme2.5_01183.1_g00010.1	2d	337	36.62	9.55
SmWRKY25	Sme2.5_01372.1_g00013.1/pseudo	2a	353	38.71	8.55
SmWRKY26	Sme2.5_01585.1_g00006.1	1	499	55.49	7.64
SmWRKY27	Sme2.5_01670.1_g00011.1	2e	353	39.38	5.64
SmWRKY28	Sme2.5_01689.1_g00004.1	2e	332	36.60	5.13
SmWRKY29	Sme2.5_02107.1_g00005.1	1	615	66.36	6.12
SmWRKY30	Sme2.5_02381.1_g00007.1	2b	534	58.28	6.44
SmWRKY31	Sme2.5_02389.1_g00002.1	2c	134	15.75	9.64
SmWRKY32	Sme2.5_02587.1_g00015.1	1	529	58.74	7.17
SmWRKY33	Sme2.5_02680.1_g00006.1	1	450	49.73	9.51
SmWRKY34	Sme2.5_02752.1_g00007.1	3	296	33.91	5.94
SmWRKY35	Sme2.5_02954.1_g00006.1	1	389	43.05	7.69
SmWRKY36	Sme2.5_03205.1_g00005.1	2b	434	47.31	6.90
SmWRKY37	Sme2.5_03353.1_g00002.1	2c	288	32.62	6.18
SmWRKY38	Sme2.5_03471.1_g00002.1	2a	182	20.83	9.45
SmWRKY41	Sme2.5_03997.1_g00004.1	2c	406	44.18	5.83
SmWRKY39	Sme2.5_03533.1_g00001.1	2e	299	33.37	5.70
SmWRKY40	Sme2.5_03980.1_g00003.1	2c	319	36.33	6.49
SmWRKY42	Sme2.5_03997.1_g00007.1	2c	448	48.81	8.75
SmWRKY43	Sme2.5_04027.1_g00002.1	3	356	39.06	6.32
SmWRKY44	Sme2.5_04190.1_g00001.1	2a	339	37.64	8.84
SmWRKY45	Sme2.5_04253.1_g00002.1	2c	227	25.60	8.20
SmWRKY46	Sme2.5_04516.1_g00005.1	2c	301	33.88	6.46
SmWRKY47	Sme2.5_04517.1_g00003.1/TE	2c	1046	116.54	6.30
SmWRKY48	Sme2.5_05222.1_g00004.1	1	505	54.91	7.60
SmWRKY49	Sme2.5_06310.1_g00004.1	2a	586	66.70	8.48
SmWRKY50	Sme2.5_06988.1_g00004.1	1	492	55.21	6.65
SmWRKY51	Sme2.5_07339.1_g00001.1	3	341	38.36	7.01
SmWRKY52	Sme2.5_08092.1_g00001.1	2b	505	55.81	6.58
SmWRKY53	Sme2.5_11773.1_g00001.1/partial	3	355	40.35	5.78
SmWRKY54	Sme2.5_14019.1_g00001.1/partial	2c	241	27.65	7.67
SmWRKY55	Sme2.5_14251.1_g00004.1/partial	2c	202	23.01	7.63
SmWRKY56	Sme2.5_15021.1_g00001.1	2d	358	40.55	9.58
SmWRKY57	Sme2.5_17732.1_g00001.1	2b	362	39.30	9.17
SmWRKY58	Sme2.5_18444.1_g00001.1/partial	2c	298	33.79	9.51

## Results

### Identification of WRKY protein family in eggplant

To identify the WRKY proteins in eggplant, BLASTP search was performed against the eggplant genome database (http://eggplant.kazusa.or.jp/) using the conserved WRKY domains in Arabidopsis. About 79 putative WRKY proteins were identified. Meanwhile, 68 putative WRKY proteins were identified using HMM (Hidden Markov Model) search program with a Hidden Markov Model (PF03106). By removing the redundant proteins and proteins without WRKY domain verified by Pfam and SMART programs, a total of 58 WRKY proteins were finally confirmed in eggplant. Obviously, the WRKY family of eggplant was smaller than those of other Solanaceae plants such as tomato (81 members) (Huang et al., 2012a; Huang et al., 2012b), pepper (*Capsicum annuum* L.) (61 members) (Cheng et al., 2016) and potato (82 members) (Liu et al., 2017a; Liu et al., 2017b).

The length of WRKY proteins in eggplant ranged from 134 to 1046 aa, with an average length of approximately 396 aa. The proteins with minimum length and maximum length were SmWRKY31 and SmWRKY47, respectively. Correspondingly, the molecular weights ranged from 15.75 kDa (SmWRKY31) to 116.54 kDa (SmWRKY47). The pIs of 58 SmWRKY proteins ranged from 5.13 to 9.72, among which 34 pIs<7 and 24 pIs>7 (Table 1) (Data S3).

### Classification and phylogenetic analysis of SmWRKY proteins

Based on the number and amino acid sequences of the conserved WRKY domains, eggplant WRKY family can be classified into three main groups or seven subgroups, which is consistent with other plants. The group I contained 13 WRKY members which had two conserved WRKY domains, while 37 members were classed into group II (6 group II a, 7 group II b, 13 group II c, 8 group II d and 5 group II e). The remaining six WRKY proteins belonged to group III (Table 1).

**Figure 1:**
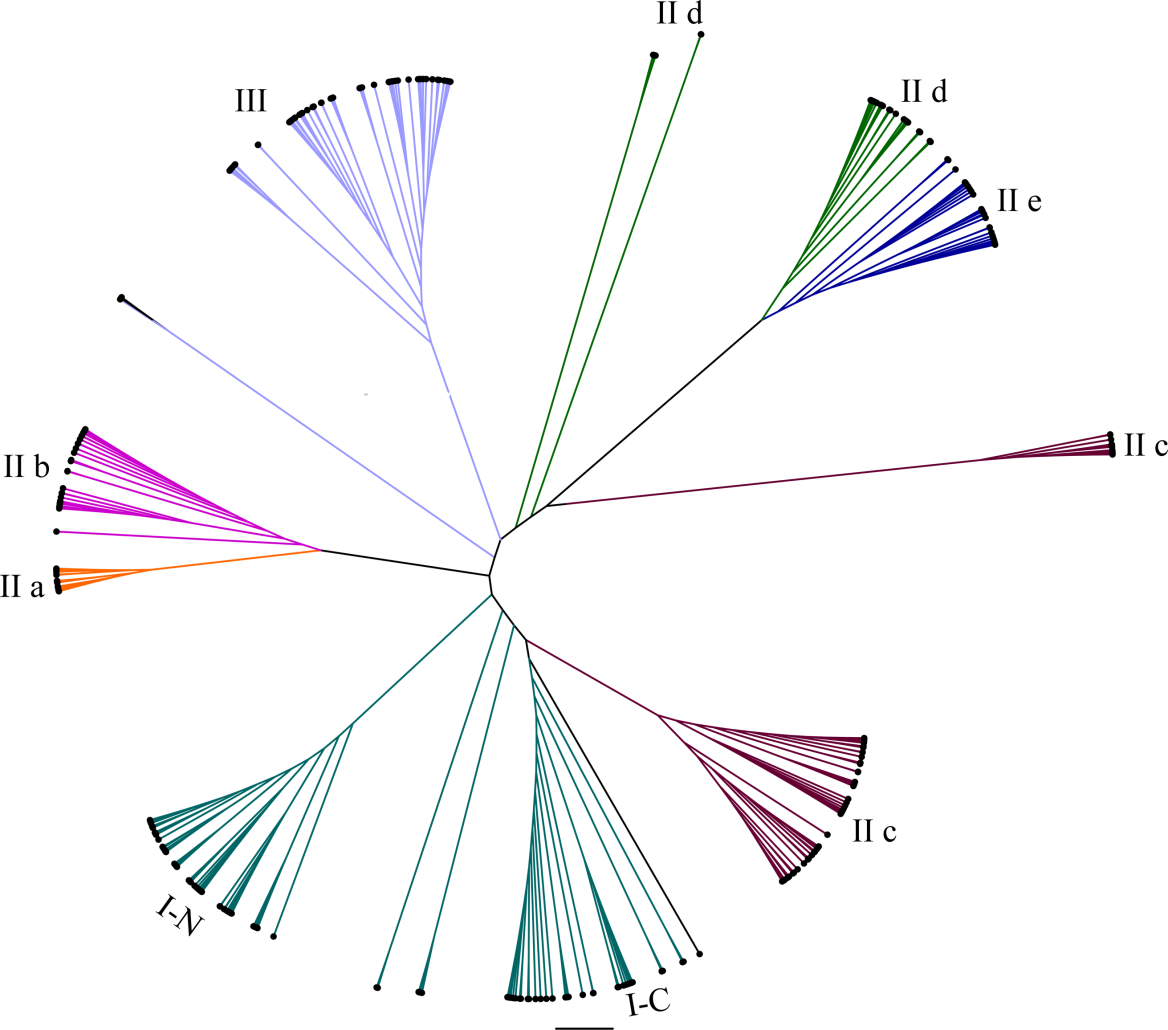
Radial phylogenetic tree of WRKY family in eggplant, tomato, Arabidopsis and rice. The conserved WRKY domain sequences of eggplant, tomato, Arabidopsis and rice were used to construct the phylogenetic tree by MEGA v5.1. The suffix ‘N’ and ‘C’ indicated the N-terminal and C-terminal WRKY domain of group I, respectively. The different groups were indicated with colors. The conserved WRKY domain sequences of eggplant, tomato, Arabidopsis and rice were used to construct the phylogenetic tree by MEGA v5.1. All the WRKY proteins were divided into three groups (I, II and III) and five subgroups (II a, II b, II c, II d and II e). The suffix ‘N’ and ‘C’ indicated the N-terminal and C-terminal WRKY domain of group I, respectively. The different groups were indicated with colors.

To study the evolutionary relationships of WRKY proteins between eggplant and other plants, a phylogenetic tree was constructed based on the conserved sequences of WRKY domains from eggplant, tomato, Arabidopsis and rice. All the WRKY proteins were divided into seven subgroups (Fig. 1). Phylogenetic analysis indicated that WRKY proteins in eggplant and tomato were closely related, compared with those in Arabidopsis and rice, such as SlWRKY54 and SmWRKY1, SlWRKY53 and SmWRKY3, SlWRKY31 and SmWRKY26, SlWRKY33 and SmWRKY32, SlWRKY3 and SmWRKY8 and so on (Data S4).

**Figure 2:**
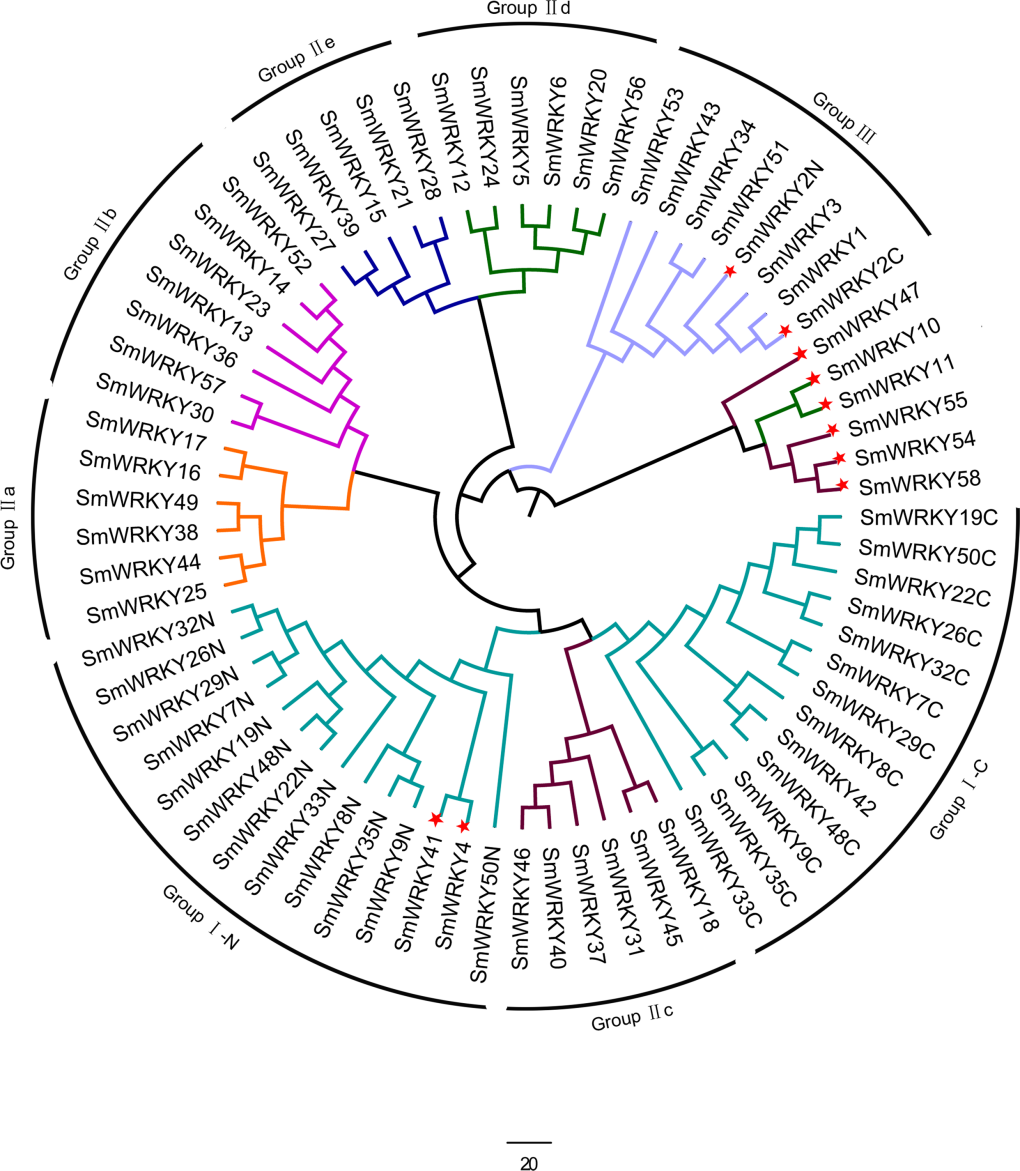
Phylogenetic tree of WRKY family in eggplant. The phylogenetic tree was constructed using the neighbor-joining method in MEGA v5.1 with the conserved WRKY domains of eggplant WRKY proteins. The eggplant WRKY proteins were grouped into three groups (I, II and III) and five subgroups (II a, II b, II c, II d and II e). The suffix ‘N’ and ‘C’ indicated the N-terminal and C-terminal WRKY domain of group I, respectively. The WRKY proteins which were not clustered into the groups they belong to were indicated with red stars. The different groups were indicated with colors.

To determine the phylogenetic relationships among the SmWRKY proteins, a phylogenetic tree was also constructed with WRKY conserved sequences using MEGAv5.1. As shown in Fig. 2, most of the SmWRKY members clustered in accordance with the classification, while SmWRKY2, SmWRKY4, SmWRKY10, SmWRKY11, SmWRKY41, SmWRKY42, SmWRKY47, SmWRKY54, SmWRKY55 and SmWRKY58 were not clustered into the corresponding branches.

### Structural analysis of WRKY variants

**Figure 3:**
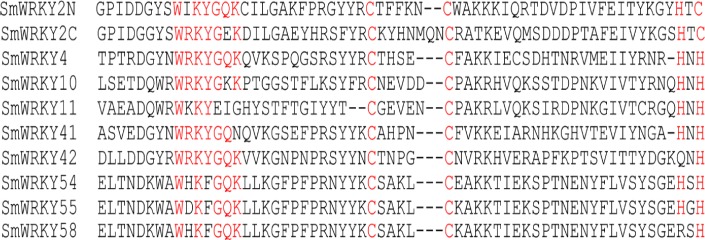
Identification of WRKY variants in eggplant. The sequences of WRKY variants were aligned. The conserved WRKYGQK and zinc-finger residues were marked in red.

The WRKY domain consists of a WRKY amino acid sequence at the N-terminal and a zinc-finger structure that is either Cx4–5Cx22–23HxH or Cx7Cx23HxC at the C-terminal. Some variants in WRKY domain were reported in the earlier studies. For example, the core sequence of WRKY domain was replaced by WRRY, WSKY, WKRY, WVKY or WKKY (Eulgem et al., 2000; Xie et al., 2005; Wei et al., 2016). The amino acids of GQK were substituted by GKK or EIG. Interestingly, WHKF and WDKF were found in eggplant WRKY proteins and GEK replaced GQK following the WRKY sequence (Fig. 3). Furthermore, it existed that the sequence of the zinc-finger motif at the C-terminal was Cx23RxH (SmWRKY58) or Cx23QxH (SmWRKY42) in eggplant WRKY proteins (Fig. 3). In SmWRKY11 protein, the sequence between the core domain (WRKYGQK) and the first cysteine of zinc-finger was two amino acids less than those normal sequences (Fig. 3). SmWRKY42 and SmWRKY58 were lack of the first histidine of conserved zinc-finger (Fig. 3). It may result in the failure of zinc-finger formation.

### Intron-exon structure and conserved motifs of *SmWRKY* genes

**Figure 4:**
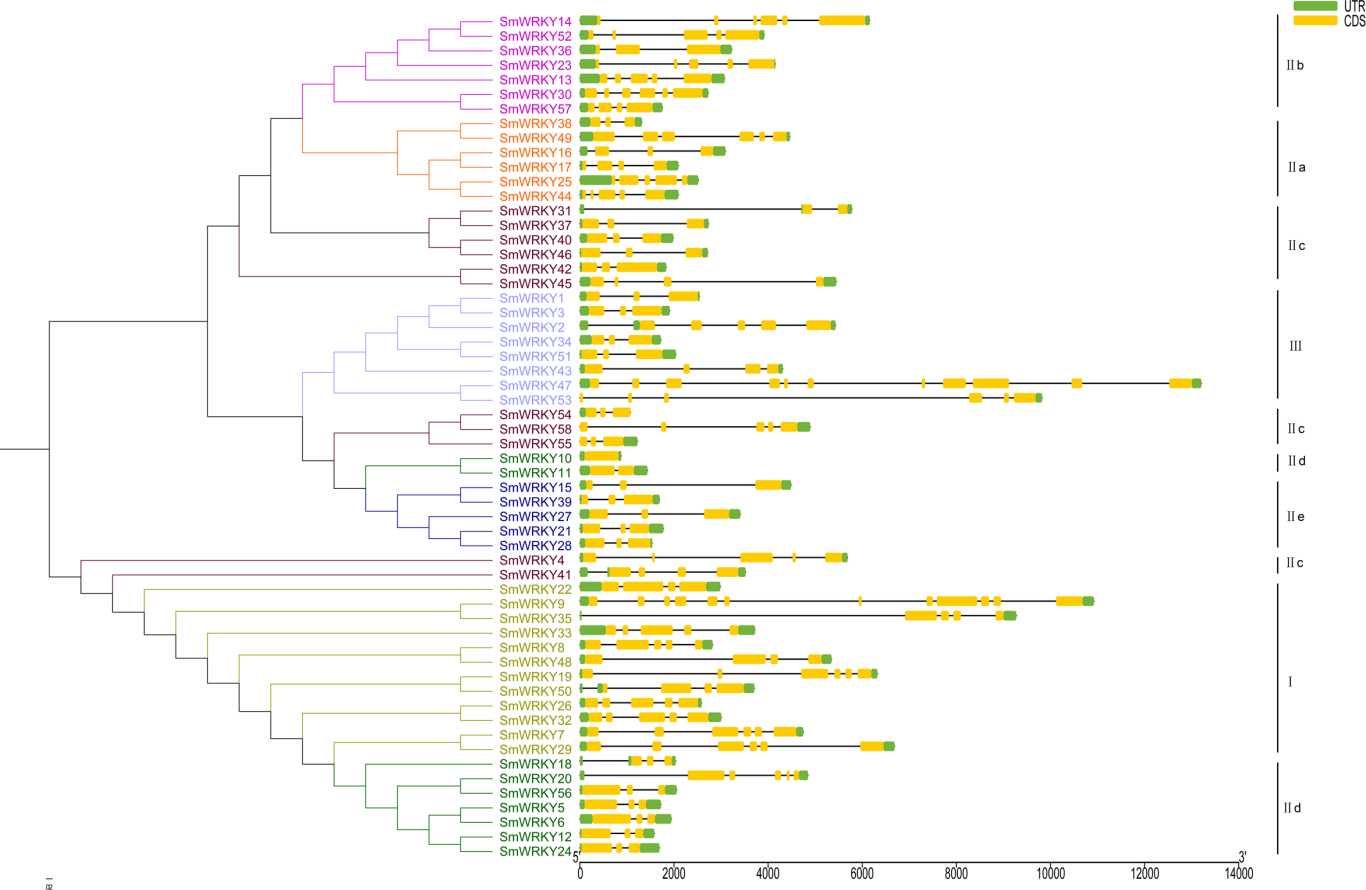
The intron-exon structure of eggplant WRKY genes according to the phylogenetic relationship. The phylogenetic tree was constructed with the full length sequences of eggplant WRKY proteins using MEGA v5.1. The intron-exon structure analysis was performed in Gene Structure Display Server. Lengths of intron and exon of each *WRKY* gene were showed in proportion. Every group was marked with different colors.

To understand the intron-exon structure of *SmWRKY* genes, we submitted the gff3 file of *SmWRKY* genes to Gene Structure Display Server (http://gsds.cbi.pku.edu.cn/) (Data S1). As shown in Fig. 4, the scope of intron number in eggplant *WRKY* family was from 0 (*SmWRKY10*) to 11 (*SmWRKY9*), with an average number of 4.4. In rice and cassava, the number of introns varied from 0 to 8 and 1 to 5, respectively (Xie et al., 2005; Wei et al., 2016). Group I contained 3-5 introns with the exception of *SmWRKY9* (11 introns). Group II a contained 3-5 introns, except for *SmWRKY38* (2 introns). Group II b contained 2–5 introns. Although several WRKY proteins were not clustered together in group II c, most of *WRKYs* contained 2 introns. The number of intron in group II d *WRKYs* was almost 2, excluding *SmWRKY10* (0 intron) and *SmWRKY20* (5 introns). All of the group II e *SmWRKYs* consisted of 2 introns. The majority of group III *WRKY* genes also included 2 introns.

**Figure 5:**
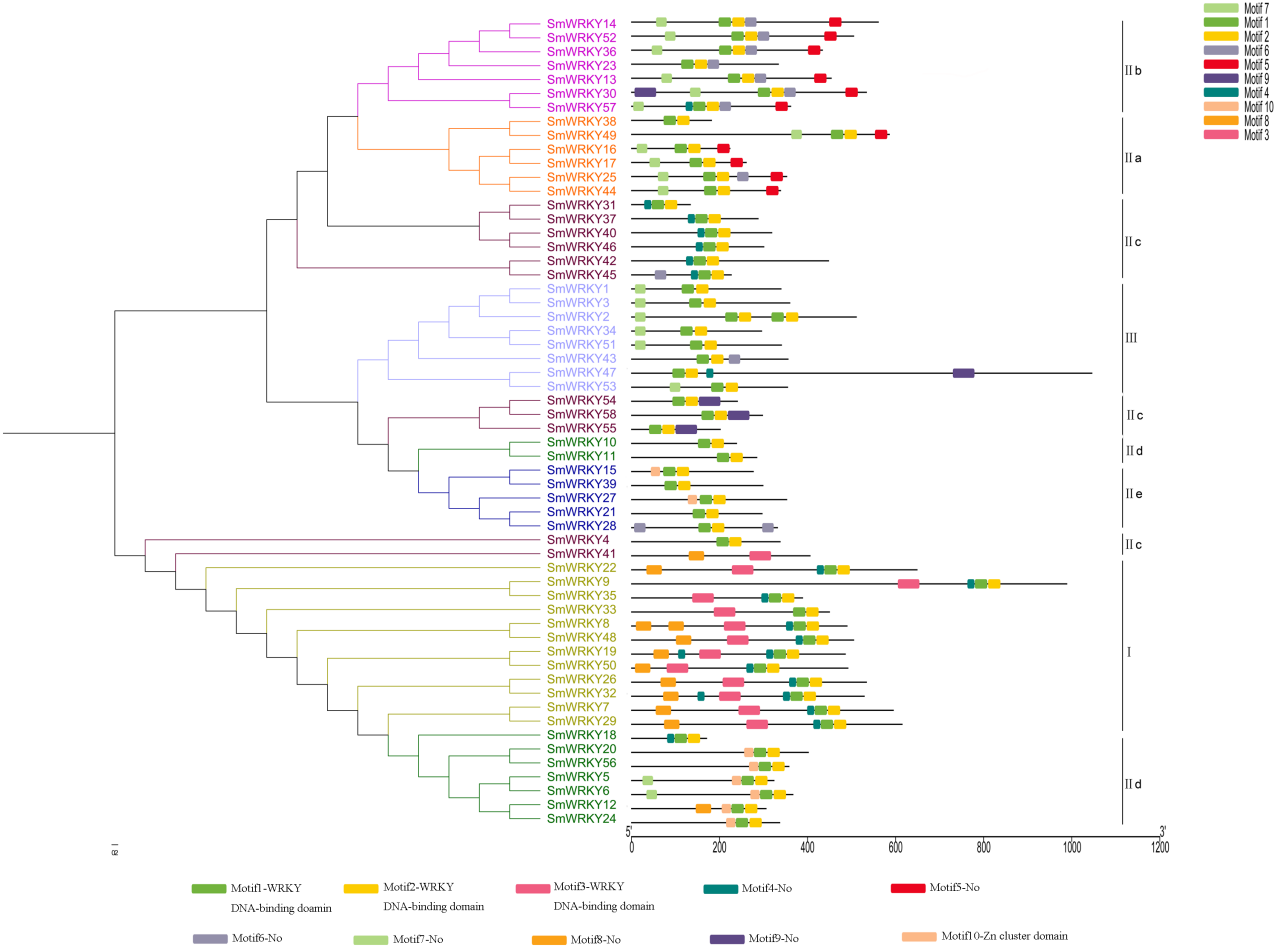
Conserved motifs of eggplant WRKY proteins according to the phylogenetic relationship. The phylogenetic tree was constructed with the full length sequences of eggplant WRKY proteins using MEGA v5.1. The conserved motifs were predicted using the MEME program. The gray line indicated the non-conserved sequences. Each conserved motif was represented by a colored box. The length of each motif was showed in proportion.

To further know the structure of eggplant WRKYs and their phylogenetic relationships, conserved motifs were analyzed using MEME tool and annotated with InterPro Scan. As we set, ten motifs were found in the sequences (Fig. 5). The first three motifs were annotated as WRKY DNA-binding domain. All the SmWRKY proteins contained two WRKY motifs at least. These results suggested that the features were conserved in the WRKY family identified from eggplant. Notably, almost the members in each group contained the same motifs. However, Yang et al. (2019) mentioned that the function of family members largely varied, even if these members were from the same clade. Therefore, these WRKY proteins classified into the same group may differentially function. Although SmWRKY18 belonged to group II c, it had the similar conserved motifs with the group II d.

### The differentially expressed *SmWRKYs* in response to cold stress

**Figure 6:**
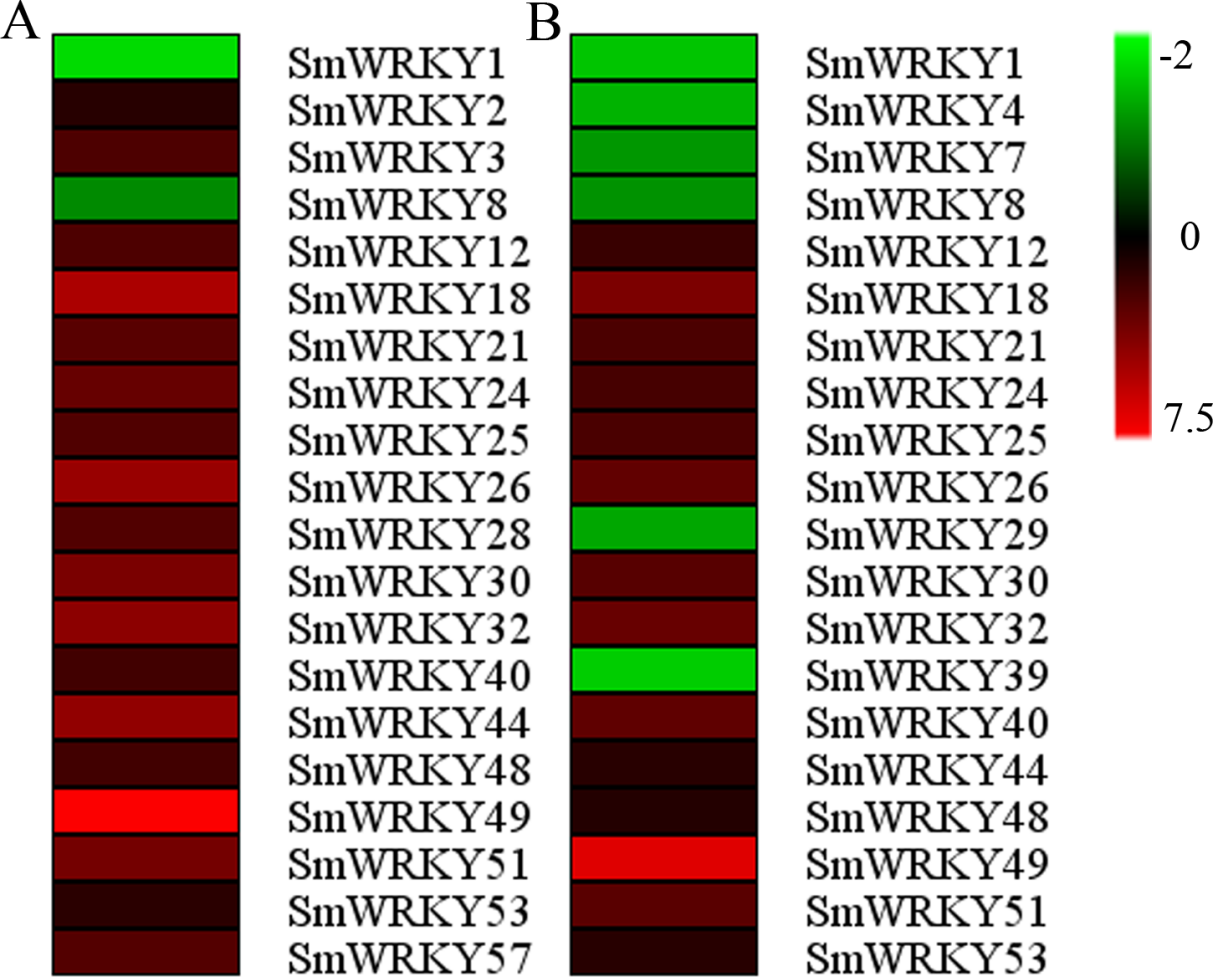
Expression profiles of the differentially expressed eggplant *WRKY* genes under cold stress. (A) The differentially expressed *WRKY* genes in sensitive cultivar after 4 °C treatment for 12 h. (B) The differentially expressed *WRKY* genes in tolerant cultivar after 4 °C treatment for 12 h. The units of the scale was Log Fold change. Green, black and red indicated down-regulated, no change and up-regualted, respectively.

Eggplant is sensitive to cold stress and often undergoes the cold stress during its growth and development, especially in the early spring (Zhou et al., 2018a; Zhou et al., 2018b). Therefore, it is important to explore the mechanism for cold tolerance and how to improve this tolerance. Previous studies have shown that WRKY transcription factors regulated the plant defense against cold stress (He et al., 2016; Zhang et al., 2016). In order to study the function of *WRKY* gene family in eggplant response to cold stress, the leaf samples from a cold-tolerant genotype and a cold-sensitive genotype were collected with the three biological replicates when the seedlings were treated for twelve hours. Then the differentially expressed *WRKY* genes in eggplant cultivars to cold stress were investigated from RNA-seq data which has been not published yet (Data S5). As shown in Fig. 6, twenty *SmWRKY* genes were identified in each cultivar. Interestingly, sixteen *WRKY* genes were common in both cultivars. Meanwhile, the sixteen *WRKY* genes showed similar expression pattern in these two cultivars when exposed to cold stress. Only *SmWRKY1* and *SmWRKY8* were blocked in the sensitive cultivar (Fig. 6A). *SmWRKY4*, *SmWRKY7*, *SmWRKY29* and *SmWRKY39* were also down-regulated in the tolerant cultivar except these two *WRKY* genes, (Fig. 6B). The transcript level of the rest of *WRKY* genes in both cultivars were enhanced. These results indicated that WRKY transcription factors could respond to cold stress and constituted a complicated network to regulate the cold tolerance in eggplant.

### Silencing *SmWRKY26* and *SmWRKY32* increased the sensitivity to cold

**Figure 7:**
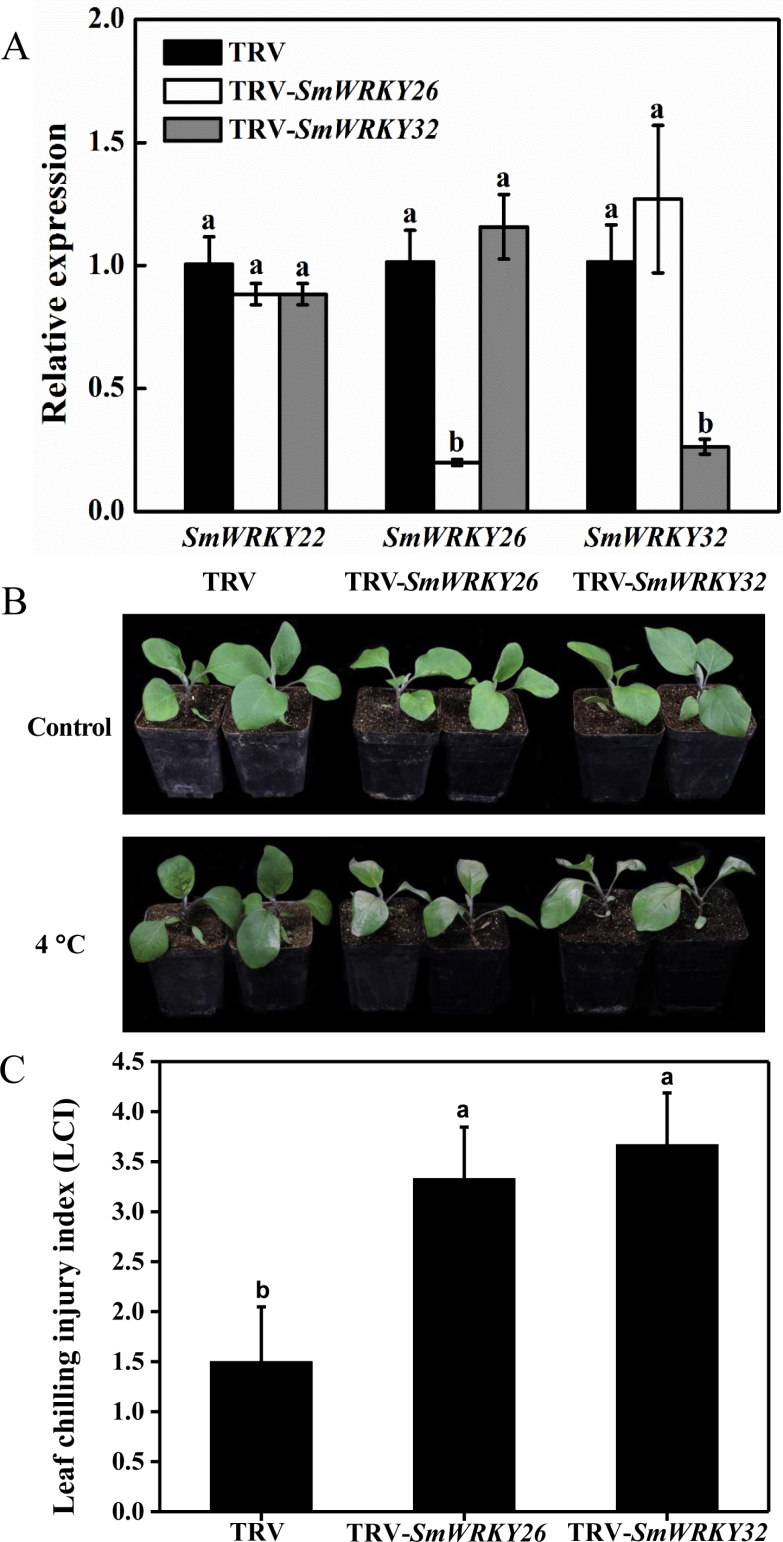
Silencing *SmWRKY26* and *SmWRKY32* enhanced the sensitivity to cold stress in eggplant. (A) The transcript level of *SmWRKY22*, *SmWRKY26* and *SmWRKY32* tested by qRT-PCR. The black, white and shaded columns represent the empty TRV control plants, SmWRKY26-silenced plants and SmWRKY32-silenced plants, respectively. Data were means ± SE calculated from three biological replicates. Values with the same letter were not significantly different according to Duncan’s multiple range tests (*P* < 0.05, *n* = 3). (B) The phenotypes of silenced plants after cold stress for five days followed by three days of recovery at 28 °C. (C) The leaf chilling injury index of VIGS-plants after cold stress. Values with the same letter were not significantly different according to Duncan’s multiple range tests (*P* < 0.05, *n* = 6). The inoculations were conducted for three independent times.

VIGS was an effective tool to study the gene functions (Zhang et al., 2018; Xu et al., 2019). VIGS mediated by TRV has been successfully employed in the functional analysis of eggplant genes (Zhou et al., 2018a; Zhou et al., 2018b). We conducted a functional validation for *SmWRKY26* and *SmWRKY32* using VIGS. The fragments of *SmWRKY26* and *SmWRKY32* were cloned into the TRV silencing vector. Subsequently, these vectors were introduced into the eggplant. Using the online VIGS tool, we found that the transcript level of *SmWRKY22* was likely affected. To detect the efficiency of silencing, the transcript level of these two *WRKY* genes was determined with qRT-PCR. As shown in Fig. 7A, the transcript level of *SmWRKY26* and *SmWRKY32* in the leaves with target gene silenced displayed a reduction of approximately 80%, compared with the leaves inoculated with the control bacterium. However, the expression of *SmWRKY22* showed no alter in any one kind of the silenced seedlings. The seedlings were transferred into 4 °C for 5 days when the fourth leaf was fully expanded. The gene-silenced seedlings showed more severe wilting and shrink, while the control seedlings presented a little chlorosis (Fig. 7B). Whereafter, the leaf chilling injury index (LCI) was calculated after treatment. The LCI of *SmWRKY26*- and *SmWRKY32-* silenced seedlings were significantly higher than that of control seedlings (Fig. 7C). The phenotypes and index upon cold stress suggested that *SmWRKY26* and *SmWRKY32* positively regulated the tolerance to cold stress in eggplant.

## Discussion

### Identification of WRKY proteins in eggplant

Characterization of gene families from eggplant genome is more and more important and imperative due to its importance for understanding the roles in plant responses to environmental stresses. In this study, we have identified 58 *WRKY* genes through searching the sequenced genome (Table 1). The *WRKY* gene family of eggplant was smaller than those of other plants such as the representative of Solanaceae plant tomato (81 *WRKY* genes). The WRKY members identified in this study was fewer than that reported in the PlantTFDB website (http://planttfdb.cbi.pku.edu.cn/family.php?sp=Sme&fam=WRKY#family_intro). 65 eggplant WRKY proteins were recorded in the plant transcription factor database. However, nine proteins either contained no WRKY feature structures, or had incomplete WRKY domains. Moreover, another two WRKY proteins were found in our study. 74 hits were obtained when “WRKY” was set as the keyword in eggplant genome. Among these, 55 hits were consistent with the WRKY proteins in our study. It lacked three WRKY proteins (Sme2.5_06310.1_g00004.1, Sme2.5_04517.1_g00003.1 and Sme2.5_00196.1_g00004.1) which were found in our study. The rest 19 predicted proteins were misannotated and listed in Data S6.

Interestingly, it was larger than that reported in Yang’s study (50 *SmWRKY* genes) (Yang et al., 2015), which might be due to that Yang identified the *WRKY* genes from transcriptome data.

### Phylogenetic tree and variant analysis of eggplant WRKY

The phylogenetic tree among the eggplant WRKY proteins has shown their relatively conserved evolutionary relationships (Fig. 2). As shown in Fig. 2, some WRKY proteins were not clustered into the corresponding branches. These WRKY proteins were identified as WRKY variants through analyzing the amino acid sequence of these proteins (Fig. 3). For example, the C-terminal WRKY domain of WRKY2 protein had the sequence similarity with group III WRKY proteins and the last amino acid in the zinc-finger of the N-terminal WRKY domain is cysteine. These results suggested that the substitutions, addition and subtraction of amino acids of WRKY domain might be responsible for the changes in clustering. Furthermore, these WRKY variants probably lose the DNA-binding activity and the zinc-finger motif can not be formed, which lead to new molecular activities, novel biology functions and a non-functional protein. We found this phenomenon in our earlier study performed in soybean (Yang et al., 2017a; Yang et al., 2017b). The phylogenetic relationship between eggplant and other plants confirmed that WRKY proteins in eggplant contained the same subgroups with other reported plants (Data S4). In addition, eggplant WRKY proteins had a highly close relationship to tomato WRKY proteins, on account of being the Solanaceae.

### Intron-exon structure and conserved motifs in eggplant WRKY

Analysis of the distribution of intron and exon is a better way to understand the phylogenetic relationship, because intron-exon structural diversity is an important part in the evolution of gene families (Wei et al., 2016). The *WRKY* genes in group I, group II a and group II b have more introns, whereas a vast majority of the rest *WRKY* genes have only two introns. It seems that eggplant WRKY proteins originate from the first group WRKY. The conserved motifs of eggplant WRKY proteins were identified using MEME tool and InterPro Scan. All the eggplant WRKY proteins contained at least two WRKY motifs with the exception of SmWRKY41. Unlike SmWRKY4, another WRKY variant in which the Cx22HxH replaced for Cx23HxH, the core sequence of the WRKY domain in SmWRKY41 was replaced by WRKYGQN. This specific variation was potentially responsible for one WRKY motif in SmWRKY41. Motif 10 was mainly included in group II d proteins except SmWRKY10 and SmWRKY11 (Fig. 5). Using InterPro Scan, motif 10 was identified as Zn-cluster domain (IPR018872) which was found associated with the WRKY domain (Babu et al., 2006). The types of conserved motifs contained in SmWRKY10 and SmWRKY11 more closely resembled those of group II e proteins, which might be one of the reasons for their clustering (Fig. 5).

### *SmWRKY* genes in response to cold stress

Cold stress is an important factor affecting the growth and development of eggplant. To a certain extent, the response of *WRKY* genes to cold stress reflects the roles of *WRKY* genes in cold tolerance of plants (He et al., 2016). For example, Zhou et al. (2008) found that *GmWRKY21* was induced by cold treatment and the transgenic plants over-expressing *GmWRKY21* exhibited enhanced tolerance. In this study, we identified 24 differentially expressed *SmWRKY* genes in the two investigated materials from the RNA-seq data (Fig. 6). Four eggplant *WRKY* genes were specially differentially expressed in any one cultivar. This indicated that the differentially expression of eight *WRKY* genes might lead to difference in cold tolerance. In the common sixteen genes, *WRKY26* and *WRKY32* which shared a high sequence-similarity displayed up-regulated under cold stress. We conducted a VIGS experiment to verify the function of these two genes in response to cold. The different phenotypes and the chilling injury index between the silenced and the control plants illuminated that *SmWRKY26* and *SmWRKY32* could positively regulate the response to cold stress (Fig. 7).

Zhou et al. (2016) identified that several genes in response against *Verticillium dahlia* were annotated as *WRKY* genes, such as *Unigene21521*, *Unigene11753*, *Unigene6502* and *Unigene20920*. *SmWRKY26* and *SmWRKY32* were the orthologous of *Unigene20920* and *Unigene6502* in cultivated eggplant, respectively. In addition, the proteins encoded by *WRKY26* and *WRKY32* had a 93% and 85% degree of similarity with *SlWRKY31* and *SlWRKY33* in tomato, respectively. SlWRKY31 and SlWRKY33 were reported as SlWRKY33A and SlWRKY33B to function in disease resistance and heat tolerance in tomato (Zhou et al., 2015). These results implied that orthologous could perform variant functions in different plants.

## Conclusions

WRKY proteins play a vital role in response to different stresses, plant growth and development. In order to understand the WRKY proteins family in eggplant, a genome-wide identification of WRKY proteins was performed in this study. 58 genes encoding eggplant WRKY proteins were identified through searching the eggplant genome, which could be classified into three groups or seven subgroups in accordance with other plants. WRKY variants were identified in eggplant. Gene structure analysis showed that the number of intron in eggplant WRKY family was from 0 to 11, with an average number of 4.4. Based on conserved motif analysis, the WRKY DNA-binding domain was found conserved in eggplant WRKY proteins. Furthermore, RNA-seq data showed that *WRKY* genes were differentially expressed in eggplant response to cold stress. Structurally related SmWRKY26 and SmWRKY32 were confirmed to positively regulate the response to cold stress in eggplant. Therefore, the two preliminarily verified genes can be used as target genes for genetic improvement in cold stress. This study provides a foundation and practical reference for the functional analysis of the *WRKY* gene family in eggplant and the genetic improvement in cold-tolerance.

##  Supplemental Information

10.7717/peerj.8777/supp-1 Data S1 The eggplant *WRKY* gene features with gff3 format

10.7717/peerj.8777/supp-2 Data S2 The specific primers for VIGS vector and qRT-PCR

10.7717/peerj.8777/supp-3 Data S3 The sequences of WRKY proteins in eggplant

10.7717/peerj.8777/supp-4 Data S4 The rectangular phylogenetic tree of WRKY family in eggplant, tomato, Arabidopsis and rice

10.7717/peerj.8777/supp-5 Data S5 The differentially expressed *WRKY* genes in sensitive and tolerant cultivars under cold stress The sheet “S0 vs S12” and “T0 vs T12” indicated the differentially expressed *WRKY* genes in sensitive and tolerant cultivars after cold stress for 12 h from the RNA-seq raw data, respectively.

10.7717/peerj.8777/supp-6 Data S6 The misannotated WRKY proteins predicted in eggplant genome

## Additional Information and Declarations

### Data Availability

The following information was supplied regarding data availability: The RNA-seq data is available at NCBI Genbank: PRJNA572318. Sequences are available in Data S3.
