# Supplementary material for: Identification of WRKY gene family and characterization of cold stress-responsive WRKY genes in eggplant
Source: PeerJ. 2020 Mar 17;8:e8777. doi: 10.7717/peerj.8777 (PMC7083166; doi:10.7717/peerj.8777)
Supplement: Data S1 [file peerj-08-8777-s001.doc]

**Data S1 The eggplant *WRKY* gene features with gff3 format**

SmWRKY1 . gene 128166 130709 0.01 + . ID=SmWRKY1.1

SmWRKY1 . mRNA 128166 130709 0.01 + . ID=SmWRKY1;Parent=SmWRKY1.1

SmWRKY1 . exon 128166 128587 . + . ID=exon:SmWRKY1;Parent=SmWRKY1

SmWRKY1 . intron 128588 129307 0.99 + . ID=intron:SmWRKY1;Parent=SmWRKY1

SmWRKY1 . exon 129308 129436 . + . ID=exon:SmWRKY1;Parent=SmWRKY1

SmWRKY1 . intron 129437 130054 0.97 + . ID=intron:SmWRKY1;Parent=SmWRKY1

SmWRKY1 . exon 130055 130709 . + . ID=exon:SmWRKY1;Parent=SmWRKY1

SmWRKY2 . gene 177136 182569 0.07 + . ID=SmWRKY2.1

SmWRKY2 . mRNA 177136 182569 0.07 + . ID=SmWRKY2;Parent=SmWRKY2.1

SmWRKY2 . exon 177136 177312 . + . ID=exon:SmWRKY2;Parent=SmWRKY2

SmWRKY2 . exon 178274 178730 . + . ID=exon:SmWRKY2;Parent=SmWRKY2

SmWRKY2 . intron 178731 179499 0.87 + . ID=intron:SmWRKY2;Parent=SmWRKY2

SmWRKY2 . exon 179500 179726 . + . ID=exon:SmWRKY2;Parent=SmWRKY2

SmWRKY2 . intron 179727 180491 0.64 + . ID=intron:SmWRKY2;Parent=SmWRKY2

SmWRKY2 . exon 180492 180648 . + . ID=exon:SmWRKY2;Parent=SmWRKY2

SmWRKY2 . intron 180649 180988 0.74 + . ID=intron:SmWRKY2;Parent=SmWRKY2

SmWRKY2 . exon 180989 181303 . + . ID=exon:SmWRKY2;Parent=SmWRKY2

SmWRKY2 . intron 181304 181948 0.97 + . ID=intron:SmWRKY2;Parent=SmWRKY2

SmWRKY2 . exon 181949 182569 . + . ID=exon:SmWRKY2;Parent=SmWRKY2

SmWRKY3 . gene 194820 196730 0.07 + . ID=SmWRKY3.1

SmWRKY3 . mRNA 194820 196730 0.07 + . ID=SmWRKY3;Parent=SmWRKY3.1

SmWRKY3 . exon 194820 195339 . + . ID=exon:SmWRKY3;Parent=SmWRKY3

SmWRKY3 . intron 195340 195673 1 + . ID=intron:SmWRKY3;Parent=SmWRKY3

SmWRKY3 . exon 195674 195802 . + . ID=exon:SmWRKY3;Parent=SmWRKY3

SmWRKY3 . intron 195803 195931 0.98 + . ID=intron:SmWRKY3;Parent=SmWRKY3

SmWRKY3 . exon 195932 196730 . + . ID=exon:SmWRKY3;Parent=SmWRKY3

SmWRKY4 . gene 245693 251382 0.1 - . ID=SmWRKY4.1

SmWRKY4 . mRNA 245693 251382 0.1 - . ID=SmWRKY4;Parent=SmWRKY4.1

SmWRKY4 . exon 245693 246169 . - . ID=exon:SmWRKY4;Parent=SmWRKY4

SmWRKY4 . intron 246170 246792 0.99 - . ID=intron:SmWRKY4;Parent=SmWRKY4

SmWRKY4 . exon 246793 246862 . - . ID=exon:SmWRKY4;Parent=SmWRKY4

SmWRKY4 . intron 246863 247283 0.53 - . ID=intron:SmWRKY4;Parent=SmWRKY4

SmWRKY4 . exon 247284 247971 . - . ID=exon:SmWRKY4;Parent=SmWRKY4

SmWRKY4 . intron 247972 249793 0.95 - . ID=intron:SmWRKY4;Parent=SmWRKY4

SmWRKY4 . exon 249794 249846 . - . ID=exon:SmWRKY4;Parent=SmWRKY4

SmWRKY4 . intron 249847 251036 0.95 - . ID=intron:SmWRKY4;Parent=SmWRKY4

SmWRKY4 . exon 251037 251382 . - . ID=exon:SmWRKY4;Parent=SmWRKY4

SmWRKY5 . gene 197639 199362 0.07 - . ID=SmWRKY5.1

SmWRKY5 . mRNA 197639 199362 0.07 - . ID=SmWRKY5;Parent=SmWRKY5.1

SmWRKY5 . exon 197639 198115 . - . ID=exon:SmWRKY5;Parent=SmWRKY5

SmWRKY5 . intron 198116 198201 0.99 - . ID=intron:SmWRKY5;Parent=SmWRKY5

SmWRKY5 . exon 198202 198327 . - . ID=exon:SmWRKY5;Parent=SmWRKY5

SmWRKY5 . intron 198328 198572 0.99 - . ID=intron:SmWRKY5;Parent=SmWRKY5

SmWRKY5 . exon 198573 199362 . - . ID=exon:SmWRKY5;Parent=SmWRKY5

SmWRKY6 . gene 166716 168661 0.09 + . ID=SmWRKY6.1

SmWRKY6 . mRNA 166716 168661 0.09 + . ID=SmWRKY6;Parent=SmWRKY6.1

SmWRKY6 . exon 166716 167801 . + . ID=exon:SmWRKY6;Parent=SmWRKY6

SmWRKY6 . intron 167802 167918 1 + . ID=intron:SmWRKY6;Parent=SmWRKY6

SmWRKY6 . exon 167919 168044 . + . ID=exon:SmWRKY6;Parent=SmWRKY6

SmWRKY6 . intron 168045 168149 1 + . ID=intron:SmWRKY6;Parent=SmWRKY6

SmWRKY6 . exon 168150 168661 . + . ID=exon:SmWRKY6;Parent=SmWRKY6

SmWRKY7 . gene 105559 110307 0.3 - . ID=SmWRKY7.1

SmWRKY7 . mRNA 105559 110307 0.3 - . ID=SmWRKY7;Parent=SmWRKY7.1

SmWRKY7 . exon 105559 106187 . - . ID=exon:SmWRKY7;Parent=SmWRKY7

SmWRKY7 . intron 106188 106438 1 - . ID=intron:SmWRKY7;Parent=SmWRKY7

SmWRKY7 . exon 106439 106594 . - . ID=exon:SmWRKY7;Parent=SmWRKY7

SmWRKY7 . intron 106595 106663 1 - . ID=intron:SmWRKY7;Parent=SmWRKY7

SmWRKY7 . exon 106664 106832 . - . ID=exon:SmWRKY7;Parent=SmWRKY7

SmWRKY7 . intron 106833 106941 0.9 - . ID=intron:SmWRKY7;Parent=SmWRKY7

SmWRKY7 . exon 106942 107494 . - . ID=exon:SmWRKY7;Parent=SmWRKY7

SmWRKY7 . intron 107495 108524 0.99 - . ID=intron:SmWRKY7;Parent=SmWRKY7

SmWRKY7 . exon 108525 108719 . - . ID=exon:SmWRKY7;Parent=SmWRKY7

SmWRKY7 . intron 108720 109907 0.98 - . ID=intron:SmWRKY7;Parent=SmWRKY7

SmWRKY7 . exon 109908 110307 . - . ID=exon:SmWRKY7;Parent=SmWRKY7

SmWRKY8 . gene 191421 194242 0.18 - . ID=SmWRKY8.1

SmWRKY8 . mRNA 191421 194242 0.18 - . ID=SmWRKY8;Parent=SmWRKY8.1

SmWRKY8 . exon 191421 191797 . - . ID=exon:SmWRKY8;Parent=SmWRKY8

SmWRKY8 . intron 191798 192274 0.81 - . ID=intron:SmWRKY8;Parent=SmWRKY8

SmWRKY8 . exon 192275 192418 . - . ID=exon:SmWRKY8;Parent=SmWRKY8

SmWRKY8 . intron 192419 192511 1 - . ID=intron:SmWRKY8;Parent=SmWRKY8

SmWRKY8 . exon 192512 192664 . - . ID=exon:SmWRKY8;Parent=SmWRKY8

SmWRKY8 . intron 192665 192778 1 - . ID=intron:SmWRKY8;Parent=SmWRKY8

SmWRKY8 . exon 192779 193467 . - . ID=exon:SmWRKY8;Parent=SmWRKY8

SmWRKY8 . intron 193468 193797 0.98 - . ID=intron:SmWRKY8;Parent=SmWRKY8

SmWRKY8 . exon 193798 194242 . - . ID=exon:SmWRKY8;Parent=SmWRKY8

SmWRKY9 . gene 13814 24737 0.05 - . ID=SmWRKY9.1

SmWRKY9 . mRNA 13814 24737 0.05 - . ID=SmWRKY9;Parent=SmWRKY9.1

SmWRKY9 . exon 13814 14619 . - . ID=exon:SmWRKY9;Parent=SmWRKY9

SmWRKY9 . intron 14620 15789 1 - . ID=intron:SmWRKY9;Parent=SmWRKY9

SmWRKY9 . exon 15790 15948 . - . ID=exon:SmWRKY9;Parent=SmWRKY9

SmWRKY9 . intron 15949 16039 1 - . ID=intron:SmWRKY9;Parent=SmWRKY9

SmWRKY9 . exon 16040 16214 . - . ID=exon:SmWRKY9;Parent=SmWRKY9

SmWRKY9 . intron 16215 16293 1 - . ID=intron:SmWRKY9;Parent=SmWRKY9

SmWRKY9 . exon 16294 17153 . - . ID=exon:SmWRKY9;Parent=SmWRKY9

SmWRKY9 . intron 17154 17235 1 - . ID=intron:SmWRKY9;Parent=SmWRKY9

SmWRKY9 . exon 17236 17370 . - . ID=exon:SmWRKY9;Parent=SmWRKY9

SmWRKY9 . intron 17371 18750 0.79 - . ID=intron:SmWRKY9;Parent=SmWRKY9

SmWRKY9 . exon 18751 18813 . - . ID=exon:SmWRKY9;Parent=SmWRKY9

SmWRKY9 . intron 18814 21553 0.67 - . ID=intron:SmWRKY9;Parent=SmWRKY9

SmWRKY9 . exon 21554 21667 . - . ID=exon:SmWRKY9;Parent=SmWRKY9

SmWRKY9 . intron 21668 21816 0.89 - . ID=intron:SmWRKY9;Parent=SmWRKY9

SmWRKY9 . exon 21817 22024 . - . ID=exon:SmWRKY9;Parent=SmWRKY9

SmWRKY9 . intron 22025 22477 0.97 - . ID=intron:SmWRKY9;Parent=SmWRKY9

SmWRKY9 . exon 22478 22716 . - . ID=exon:SmWRKY9;Parent=SmWRKY9

SmWRKY9 . intron 22717 22821 1 - . ID=intron:SmWRKY9;Parent=SmWRKY9

SmWRKY9 . exon 22822 22943 . - . ID=exon:SmWRKY9;Parent=SmWRKY9

SmWRKY9 . intron 22944 23362 0.96 - . ID=intron:SmWRKY9;Parent=SmWRKY9

SmWRKY9 . exon 23363 23507 . - . ID=exon:SmWRKY9;Parent=SmWRKY9

SmWRKY9 . intron 23508 24363 0.91 - . ID=intron:SmWRKY9;Parent=SmWRKY9

SmWRKY9 . exon 24364 24737 . - . ID=exon:SmWRKY9;Parent=SmWRKY9

SmWRKY10 . gene 135076 135954 0.47 + . ID=SmWRKY10.1

SmWRKY10 . mRNA 135076 135954 0.47 + . ID=SmWRKY10;Parent=SmWRKY10.1

SmWRKY10 . exon 135076 135954 . + . ID=exon:SmWRKY10;Parent=SmWRKY10

SmWRKY11 . gene 136606 138043 0.03 + . ID=SmWRKY11.1

SmWRKY11 . mRNA 136606 138043 0.03 + . ID=SmWRKY11;Parent=SmWRKY11.1

SmWRKY11 . exon 136606 137346 . + . ID=exon:SmWRKY11;Parent=SmWRKY11

SmWRKY11 . intron 137347 137417 0.98 + . ID=intron:SmWRKY11;Parent=SmWRKY11

SmWRKY11 . exon 137418 138043 . + . ID=exon:SmWRKY11;Parent=SmWRKY11

SmWRKY12 . gene 97774 99357 0.05 - . ID=SmWRKY12.1

SmWRKY12 . mRNA 97774 99357 0.05 - . ID=SmWRKY12;Parent=SmWRKY12.1

SmWRKY12 . exon 97774 98182 . - . ID=exon:SmWRKY12;Parent=SmWRKY12

SmWRKY12 . intron 98183 98280 1 - . ID=intron:SmWRKY12;Parent=SmWRKY12

SmWRKY12 . exon 98281 98406 . - . ID=exon:SmWRKY12;Parent=SmWRKY12

SmWRKY12 . intron 98407 98703 1 - . ID=intron:SmWRKY12;Parent=SmWRKY12

SmWRKY12 . exon 98704 99357 . - . ID=exon:SmWRKY12;Parent=SmWRKY12

SmWRKY13 . gene 109486 112563 0.11 + . ID=SmWRKY13.1

SmWRKY13 . mRNA 109486 112563 0.11 + . ID=SmWRKY13;Parent=SmWRKY13.1

SmWRKY13 . exon 109486 110070 . + . ID=exon:SmWRKY13;Parent=SmWRKY13

SmWRKY13 . intron 110071 110229 0.98 + . ID=intron:SmWRKY13;Parent=SmWRKY13

SmWRKY13 . exon 110230 110361 . + . ID=exon:SmWRKY13;Parent=SmWRKY13

SmWRKY13 . intron 110362 110566 0.99 + . ID=intron:SmWRKY13;Parent=SmWRKY13

SmWRKY13 . exon 110567 110929 . + . ID=exon:SmWRKY13;Parent=SmWRKY13

SmWRKY13 . intron 110930 111014 0.98 + . ID=intron:SmWRKY13;Parent=SmWRKY13

SmWRKY13 . exon 111015 111131 . + . ID=exon:SmWRKY13;Parent=SmWRKY13

SmWRKY13 . intron 111132 111692 0.95 + . ID=intron:SmWRKY13;Parent=SmWRKY13

SmWRKY13 . exon 111693 112563 . + . ID=exon:SmWRKY13;Parent=SmWRKY13

SmWRKY14 . gene 55438 61597 0.06 - . ID=SmWRKY14.1

SmWRKY14 . mRNA 55438 61597 0.06 - . ID=SmWRKY14;Parent=SmWRKY14.1

SmWRKY14 . exon 55438 56513 . - . ID=exon:SmWRKY14;Parent=SmWRKY14

SmWRKY14 . intron 56514 57185 0.81 - . ID=intron:SmWRKY14;Parent=SmWRKY14

SmWRKY14 . exon 57186 57299 . - . ID=exon:SmWRKY14;Parent=SmWRKY14

SmWRKY14 . intron 57300 57403 0.87 - . ID=intron:SmWRKY14;Parent=SmWRKY14

SmWRKY14 . exon 57404 57758 . - . ID=exon:SmWRKY14;Parent=SmWRKY14

SmWRKY14 . intron 57759 57834 0.4 - . ID=intron:SmWRKY14;Parent=SmWRKY14

SmWRKY14 . exon 57835 57914 . - . ID=exon:SmWRKY14;Parent=SmWRKY14

SmWRKY14 . intron 57915 58652 0.7 - . ID=intron:SmWRKY14;Parent=SmWRKY14

SmWRKY14 . exon 58653 58742 . - . ID=exon:SmWRKY14;Parent=SmWRKY14

SmWRKY14 . intron 58743 61157 0.92 - . ID=intron:SmWRKY14;Parent=SmWRKY14

SmWRKY14 . exon 61158 61597 . - . ID=exon:SmWRKY14;Parent=SmWRKY14

SmWRKY15 . gene 91276 95765 0.15 + . ID=SmWRKY15.1

SmWRKY15 . mRNA 91276 95765 0.15 + . ID=SmWRKY15;Parent=SmWRKY15.1

SmWRKY15 . exon 91276 91549 . + . ID=exon:SmWRKY15;Parent=SmWRKY15

SmWRKY15 . intron 91550 92127 0.73 + . ID=intron:SmWRKY15;Parent=SmWRKY15

SmWRKY15 . exon 92128 92271 . + . ID=exon:SmWRKY15;Parent=SmWRKY15

SmWRKY15 . intron 92272 95004 0.96 + . ID=intron:SmWRKY15;Parent=SmWRKY15

SmWRKY15 . exon 95005 95765 . + . ID=exon:SmWRKY15;Parent=SmWRKY15

SmWRKY16 . gene 133016 136111 0.09 + . ID=SmWRKY16.1

SmWRKY16 . mRNA 133016 136111 0.09 + . ID=SmWRKY16;Parent=SmWRKY16.1

SmWRKY16 . exon 133016 133177 . + . ID=exon:SmWRKY16;Parent=SmWRKY16

SmWRKY16 . exon 133344 133640 . + . ID=exon:SmWRKY16;Parent=SmWRKY16

SmWRKY16 . intron 133641 134454 1 + . ID=intron:SmWRKY16;Parent=SmWRKY16

SmWRKY16 . exon 134455 134571 . + . ID=exon:SmWRKY16;Parent=SmWRKY16

SmWRKY16 . intron 134572 135580 0.59 + . ID=intron:SmWRKY16;Parent=SmWRKY16

SmWRKY16 . exon 135581 136111 . + . ID=exon:SmWRKY16;Parent=SmWRKY16

SmWRKY17 . gene 160796 162890 0.17 + . ID=SmWRKY17.1

SmWRKY17 . mRNA 160796 162890 0.17 + . ID=SmWRKY17;Parent=SmWRKY17.1

SmWRKY17 . exon 160796 160931 . + . ID=exon:SmWRKY17;Parent=SmWRKY17

SmWRKY17 . intron 160932 161170 0.77 + . ID=intron:SmWRKY17;Parent=SmWRKY17

SmWRKY17 . exon 161171 161482 . + . ID=exon:SmWRKY17;Parent=SmWRKY17

SmWRKY17 . intron 161483 161614 0.99 + . ID=intron:SmWRKY17;Parent=SmWRKY17

SmWRKY17 . exon 161615 161731 . + . ID=exon:SmWRKY17;Parent=SmWRKY17

SmWRKY17 . intron 161732 162363 0.99 + . ID=intron:SmWRKY17;Parent=SmWRKY17

SmWRKY17 . exon 162364 162890 . + . ID=exon:SmWRKY17;Parent=SmWRKY17

SmWRKY18 . gene 64056 66092 0.09 + . ID=SmWRKY18.1

SmWRKY18 . mRNA 64056 66092 0.09 + . ID=SmWRKY18;Parent=SmWRKY18.1

SmWRKY18 . exon 64056 64116 . + . ID=exon:SmWRKY18;Parent=SmWRKY18

SmWRKY18 . exon 65088 65371 . + . ID=exon:SmWRKY18;Parent=SmWRKY18

SmWRKY18 . intron 65372 65478 1 + . ID=intron:SmWRKY18;Parent=SmWRKY18

SmWRKY18 . exon 65479 65617 . + . ID=exon:SmWRKY18;Parent=SmWRKY18

SmWRKY18 . intron 65618 65852 0.97 + . ID=intron:SmWRKY18;Parent=SmWRKY18

SmWRKY18 . exon 65853 66092 . + . ID=exon:SmWRKY18;Parent=SmWRKY18

SmWRKY19 . gene 43212 49537 0.04 - . ID=SmWRKY19.1

SmWRKY19 . mRNA 43212 49537 0.04 - . ID=SmWRKY19;Parent=SmWRKY19.1

SmWRKY19 . exon 43212 43635 . - . ID=exon:SmWRKY19;Parent=SmWRKY19

SmWRKY19 . intron 43636 43757 0.17 - . ID=intron:SmWRKY19;Parent=SmWRKY19

SmWRKY19 . exon 43758 43894 . - . ID=exon:SmWRKY19;Parent=SmWRKY19

SmWRKY19 . intron 43895 43996 0.28 - . ID=intron:SmWRKY19;Parent=SmWRKY19

SmWRKY19 . exon 43997 44131 . - . ID=exon:SmWRKY19;Parent=SmWRKY19

SmWRKY19 . intron 44132 44259 0.98 - . ID=intron:SmWRKY19;Parent=SmWRKY19

SmWRKY19 . exon 44260 44834 . - . ID=exon:SmWRKY19;Parent=SmWRKY19

SmWRKY19 . intron 44835 46510 0.93 - . ID=intron:SmWRKY19;Parent=SmWRKY19

SmWRKY19 . exon 46511 46609 . - . ID=exon:SmWRKY19;Parent=SmWRKY19

SmWRKY19 . intron 46610 49258 0.93 - . ID=intron:SmWRKY19;Parent=SmWRKY19

SmWRKY19 . exon 49259 49537 . - . ID=exon:SmWRKY19;Parent=SmWRKY19

SmWRKY20 . gene 90326 95174 0.02 + . ID=SmWRKY20.1

SmWRKY20 . mRNA 90326 95174 0.02 + . ID=SmWRKY20;Parent=SmWRKY20.1

SmWRKY20 . exon 90326 90422 . + . ID=exon:SmWRKY20;Parent=SmWRKY20

SmWRKY20 . exon 92620 93399 . + . ID=exon:SmWRKY20;Parent=SmWRKY20

SmWRKY20 . intron 93400 93500 1 + . ID=intron:SmWRKY20;Parent=SmWRKY20

SmWRKY20 . exon 93501 93626 . + . ID=exon:SmWRKY20;Parent=SmWRKY20

SmWRKY20 . intron 93627 94467 0.96 + . ID=intron:SmWRKY20;Parent=SmWRKY20

SmWRKY20 . exon 94468 94600 . + . ID=exon:SmWRKY20;Parent=SmWRKY20

SmWRKY20 . intron 94601 94721 0.19 + . ID=intron:SmWRKY20;Parent=SmWRKY20

SmWRKY20 . exon 94722 94782 . + . ID=exon:SmWRKY20;Parent=SmWRKY20

SmWRKY20 . intron 94783 94867 0.29 + . ID=intron:SmWRKY20;Parent=SmWRKY20

SmWRKY20 . exon 94868 95174 . + . ID=exon:SmWRKY20;Parent=SmWRKY20

SmWRKY21 . gene 12086 13863 0.28 + . ID=SmWRKY21.1

SmWRKY21 . mRNA 12086 13863 0.28 + . ID=SmWRKY21;Parent=SmWRKY21.1

SmWRKY21 . exon 12086 12518 . + . ID=exon:SmWRKY21;Parent=SmWRKY21

SmWRKY21 . intron 12519 12947 1 + . ID=intron:SmWRKY21;Parent=SmWRKY21

SmWRKY21 . exon 12948 13058 . + . ID=exon:SmWRKY21;Parent=SmWRKY21

SmWRKY21 . intron 13059 13142 0.99 + . ID=intron:SmWRKY21;Parent=SmWRKY21

SmWRKY21 . exon 13143 13863 . + . ID=exon:SmWRKY21;Parent=SmWRKY21

SmWRKY22 . gene 63961 66947 0.16 - . ID=SmWRKY22.1

SmWRKY22 . mRNA 63961 66947 0.16 - . ID=SmWRKY22;Parent=SmWRKY22.1

SmWRKY22 . exon 63961 64836 . - . ID=exon:SmWRKY22;Parent=SmWRKY22

SmWRKY22 . intron 64837 64917 1 - . ID=intron:SmWRKY22;Parent=SmWRKY22

SmWRKY22 . exon 64918 65076 . - . ID=exon:SmWRKY22;Parent=SmWRKY22

SmWRKY22 . intron 65077 65175 1 - . ID=intron:SmWRKY22;Parent=SmWRKY22

SmWRKY22 . exon 65176 66029 . - . ID=exon:SmWRKY22;Parent=SmWRKY22

SmWRKY22 . intron 66030 66119 0.92 - . ID=intron:SmWRKY22;Parent=SmWRKY22

SmWRKY22 . exon 66120 66947 . - . ID=exon:SmWRKY22;Parent=SmWRKY22

SmWRKY23 . gene 8484 12637 0.05 - . ID=SmWRKY23.1

SmWRKY23 . mRNA 8484 12637 0.05 - . ID=SmWRKY23;Parent=SmWRKY23.1

SmWRKY23 . exon 8484 9055 . - . ID=exon:SmWRKY23;Parent=SmWRKY23

SmWRKY23 . intron 9056 9385 0.65 - . ID=intron:SmWRKY23;Parent=SmWRKY23

SmWRKY23 . exon 9386 9499 . - . ID=exon:SmWRKY23;Parent=SmWRKY23

SmWRKY23 . intron 9500 10117 0.52 - . ID=intron:SmWRKY23;Parent=SmWRKY23

SmWRKY23 . exon 10118 10314 . - . ID=exon:SmWRKY23;Parent=SmWRKY23

SmWRKY23 . intron 10315 10566 0.18 - . ID=intron:SmWRKY23;Parent=SmWRKY23

SmWRKY23 . exon 10567 10639 . - . ID=exon:SmWRKY23;Parent=SmWRKY23

SmWRKY23 . intron 10640 12229 0.32 - . ID=intron:SmWRKY23;Parent=SmWRKY23

SmWRKY23 . exon 12230 12637 . - . ID=exon:SmWRKY23;Parent=SmWRKY23

SmWRKY24 . gene 89966 91657 0.25 - . ID=SmWRKY24.1

SmWRKY24 . mRNA 89966 91657 0.25 - . ID=SmWRKY24;Parent=SmWRKY24.1

SmWRKY24 . exon 89966 90618 . - . ID=exon:SmWRKY24;Parent=SmWRKY24

SmWRKY24 . intron 90619 90758 1 - . ID=intron:SmWRKY24;Parent=SmWRKY24

SmWRKY24 . exon 90759 90884 . - . ID=exon:SmWRKY24;Parent=SmWRKY24

SmWRKY24 . intron 90885 90971 0.99 - . ID=intron:SmWRKY24;Parent=SmWRKY24

SmWRKY24 . exon 90972 91657 . - . ID=exon:SmWRKY24;Parent=SmWRKY24

SmWRKY25 . gene 80219 82737 0.09 - . ID=SmWRKY25.1

SmWRKY25 . mRNA 80219 82737 0.09 - . ID=SmWRKY25;Parent=SmWRKY25.1

SmWRKY25 . exon 80219 80568 . - . ID=exon:SmWRKY25;Parent=SmWRKY25

SmWRKY25 . intron 80569 80674 0.54 - . ID=intron:SmWRKY25;Parent=SmWRKY25

SmWRKY25 . exon 80675 81132 . - . ID=exon:SmWRKY25;Parent=SmWRKY25

SmWRKY25 . intron 81133 81245 1 - . ID=intron:SmWRKY25;Parent=SmWRKY25

SmWRKY25 . exon 81246 81362 . - . ID=exon:SmWRKY25;Parent=SmWRKY25

SmWRKY25 . intron 81363 81491 1 - . ID=intron:SmWRKY25;Parent=SmWRKY25

SmWRKY25 . exon 81492 81902 . - . ID=exon:SmWRKY25;Parent=SmWRKY25

SmWRKY25 . intron 81903 81984 0.99 - . ID=intron:SmWRKY25;Parent=SmWRKY25

SmWRKY25 . exon 81985 82737 . - . ID=exon:SmWRKY25;Parent=SmWRKY25

SmWRKY26 . gene 69326 71913 0.15 + . ID=SmWRKY26.1

SmWRKY26 . mRNA 69326 71913 0.15 + . ID=SmWRKY26;Parent=SmWRKY26.1

SmWRKY26 . exon 69326 69713 . + . ID=exon:SmWRKY26;Parent=SmWRKY26

SmWRKY26 . intron 69714 69800 1 + . ID=intron:SmWRKY26;Parent=SmWRKY26

SmWRKY26 . exon 69801 69971 . + . ID=exon:SmWRKY26;Parent=SmWRKY26

SmWRKY26 . intron 69972 70413 0.86 + . ID=intron:SmWRKY26;Parent=SmWRKY26

SmWRKY26 . exon 70414 70889 . + . ID=exon:SmWRKY26;Parent=SmWRKY26

SmWRKY26 . intron 70890 71131 1 + . ID=intron:SmWRKY26;Parent=SmWRKY26

SmWRKY26 . exon 71132 71296 . + . ID=exon:SmWRKY26;Parent=SmWRKY26

SmWRKY26 . intron 71297 71418 0.98 + . ID=intron:SmWRKY26;Parent=SmWRKY26

SmWRKY26 . exon 71419 71913 . + . ID=exon:SmWRKY26;Parent=SmWRKY26

SmWRKY27 . gene 104136 107545 0.33 + . ID=SmWRKY27.1

SmWRKY27 . mRNA 104136 107545 0.33 + . ID=SmWRKY27;Parent=SmWRKY27.1

SmWRKY27 . exon 104136 104728 . + . ID=exon:SmWRKY27;Parent=SmWRKY27

SmWRKY27 . intron 104729 105445 0.98 + . ID=intron:SmWRKY27;Parent=SmWRKY27

SmWRKY27 . exon 105446 105586 . + . ID=exon:SmWRKY27;Parent=SmWRKY27

SmWRKY27 . intron 105587 106775 0.92 + . ID=intron:SmWRKY27;Parent=SmWRKY27

SmWRKY27 . exon 106776 107545 . + . ID=exon:SmWRKY27;Parent=SmWRKY27

SmWRKY28 . gene 41916 43453 0.26 + . ID=SmWRKY28.1

SmWRKY28 . mRNA 41916 43453 0.26 + . ID=SmWRKY28;Parent=SmWRKY28.1

SmWRKY28 . exon 41916 42439 . + . ID=exon:SmWRKY28;Parent=SmWRKY28

SmWRKY28 . intron 42440 42693 0.97 + . ID=intron:SmWRKY28;Parent=SmWRKY28

SmWRKY28 . exon 42694 42804 . + . ID=exon:SmWRKY28;Parent=SmWRKY28

SmWRKY28 . intron 42805 42935 0.98 + . ID=intron:SmWRKY28;Parent=SmWRKY28

SmWRKY28 . exon 42936 43453 . + . ID=exon:SmWRKY28;Parent=SmWRKY28

SmWRKY29 . gene 43380 50067 0.13 - . ID=SmWRKY29.1

SmWRKY29 . mRNA 43380 50067 0.13 - . ID=SmWRKY29;Parent=SmWRKY29.1

SmWRKY29 . exon 43380 44108 . - . ID=exon:SmWRKY29;Parent=SmWRKY29

SmWRKY29 . intron 44109 46077 1 - . ID=intron:SmWRKY29;Parent=SmWRKY29

SmWRKY29 . exon 46078 46233 . - . ID=exon:SmWRKY29;Parent=SmWRKY29

SmWRKY29 . intron 46234 46300 1 - . ID=intron:SmWRKY29;Parent=SmWRKY29

SmWRKY29 . exon 46301 46457 . - . ID=exon:SmWRKY29;Parent=SmWRKY29

SmWRKY29 . intron 46458 46581 0.76 - . ID=intron:SmWRKY29;Parent=SmWRKY29

SmWRKY29 . exon 46582 47131 . - . ID=exon:SmWRKY29;Parent=SmWRKY29

SmWRKY29 . intron 47132 48332 0.94 - . ID=intron:SmWRKY29;Parent=SmWRKY29

SmWRKY29 . exon 48333 48527 . - . ID=exon:SmWRKY29;Parent=SmWRKY29

SmWRKY29 . intron 48528 49620 0.8 - . ID=intron:SmWRKY29;Parent=SmWRKY29

SmWRKY29 . exon 49621 50067 . - . ID=exon:SmWRKY29;Parent=SmWRKY29

SmWRKY30 . gene 37936 40665 0.06 + . ID=SmWRKY30.1

SmWRKY30 . mRNA 37936 40665 0.06 + . ID=SmWRKY30;Parent=SmWRKY30.1

SmWRKY30 . exon 37936 38297 . + . ID=exon:SmWRKY30;Parent=SmWRKY30

SmWRKY30 . intron 38298 38451 0.99 + . ID=intron:SmWRKY30;Parent=SmWRKY30

SmWRKY30 . exon 38452 38556 . + . ID=exon:SmWRKY30;Parent=SmWRKY30

SmWRKY30 . intron 38557 38835 0.93 + . ID=intron:SmWRKY30;Parent=SmWRKY30

SmWRKY30 . exon 38836 39012 . + . ID=exon:SmWRKY30;Parent=SmWRKY30

SmWRKY30 . intron 39013 39207 0.99 + . ID=intron:SmWRKY30;Parent=SmWRKY30

SmWRKY30 . exon 39208 39534 . + . ID=exon:SmWRKY30;Parent=SmWRKY30

SmWRKY30 . intron 39535 39689 0.98 + . ID=intron:SmWRKY30;Parent=SmWRKY30

SmWRKY30 . exon 39690 39803 . + . ID=exon:SmWRKY30;Parent=SmWRKY30

SmWRKY30 . intron 39804 39912 1 + . ID=intron:SmWRKY30;Parent=SmWRKY30

SmWRKY30 . exon 39913 40665 . + . ID=exon:SmWRKY30;Parent=SmWRKY30

SmWRKY31 . gene 8826 14607 0.05 + . ID=SmWRKY31.1

SmWRKY31 . mRNA 8826 14607 0.05 + . ID=SmWRKY31;Parent=SmWRKY31.1

SmWRKY31 . exon 8826 8918 . + . ID=exon:SmWRKY31;Parent=SmWRKY31

SmWRKY31 . exon 13534 13760 . + . ID=exon:SmWRKY31;Parent=SmWRKY31

SmWRKY31 . intron 13761 14308 0.92 + . ID=intron:SmWRKY31;Parent=SmWRKY31

SmWRKY31 . exon 14309 14607 . + . ID=exon:SmWRKY31;Parent=SmWRKY31

SmWRKY32 . gene 126936 129941 0.03 + . ID=SmWRKY32.1

SmWRKY32 . mRNA 126936 129941 0.03 + . ID=SmWRKY32;Parent=SmWRKY32.1

SmWRKY32 . exon 126936 127413 . + . ID=exon:SmWRKY32;Parent=SmWRKY32

SmWRKY32 . intron 127414 127490 1 + . ID=intron:SmWRKY32;Parent=SmWRKY32

SmWRKY32 . exon 126936 127413 . + . ID=exon:SmWRKY32;Parent=SmWRKY32

SmWRKY32 . intron 127414 127490 1 + . ID=intron:SmWRKY32;Parent=SmWRKY32

SmWRKY32 . exon 127491 127631 . + . ID=exon:SmWRKY32;Parent=SmWRKY32

SmWRKY32 . intron 127632 128191 0.99 + . ID=intron:SmWRKY32;Parent=SmWRKY32

SmWRKY32 . exon 128192 128748 . + . ID=exon:SmWRKY32;Parent=SmWRKY32

SmWRKY32 . intron 128749 128839 1 + . ID=intron:SmWRKY32;Parent=SmWRKY32

SmWRKY32 . exon 128840 128998 . + . ID=exon:SmWRKY32;Parent=SmWRKY32

SmWRKY32 . intron 128999 129222 1 + . ID=intron:SmWRKY32;Parent=SmWRKY32

SmWRKY32 . exon 129223 129941 . + . ID=exon:SmWRKY32;Parent=SmWRKY32

SmWRKY33 . gene 43976 47697 0.03 + . ID=SmWRKY33.1

SmWRKY33 . mRNA 43976 47697 0.03 + . ID=SmWRKY33;Parent=SmWRKY33.1

SmWRKY33 . exon 43976 44742 . + . ID=exon:SmWRKY33;Parent=SmWRKY33

SmWRKY33 . intron 44743 44887 0.71 + . ID=intron:SmWRKY33;Parent=SmWRKY33

SmWRKY33 . exon 44888 45001 . + . ID=exon:SmWRKY33;Parent=SmWRKY33

SmWRKY33 . intron 45002 45268 0.69 + . ID=intron:SmWRKY33;Parent=SmWRKY33

SmWRKY33 . exon 45269 45948 . + . ID=exon:SmWRKY33;Parent=SmWRKY33

SmWRKY33 . intron 45949 46190 1 + . ID=intron:SmWRKY33;Parent=SmWRKY33

SmWRKY33 . exon 46191 46346 . + . ID=exon:SmWRKY33;Parent=SmWRKY33

SmWRKY33 . intron 46347 47155 0.96 + . ID=intron:SmWRKY33;Parent=SmWRKY33

SmWRKY33 . exon 47156 47697 . + . ID=exon:SmWRKY33;Parent=SmWRKY33

SmWRKY34 . gene 32274 33997 0.26 - . ID=SmWRKY34.1

SmWRKY34 . mRNA 32274 33997 0.26 - . ID=SmWRKY34;Parent=SmWRKY34.1

SmWRKY34 . exon 32274 32962 . - . ID=exon:SmWRKY34;Parent=SmWRKY34

SmWRKY34 . intron 32963 33253 0.99 - . ID=intron:SmWRKY34;Parent=SmWRKY34

SmWRKY34 . exon 33254 33382 . - . ID=exon:SmWRKY34;Parent=SmWRKY34

SmWRKY34 . intron 33383 33476 1 - . ID=intron:SmWRKY34;Parent=SmWRKY34

SmWRKY34 . exon 33477 33997 . - . ID=exon:SmWRKY34;Parent=SmWRKY34

SmWRKY35 . gene 72702 81977 0.02 - . ID=SmWRKY35.1

SmWRKY35 . mRNA 72702 81977 0.02 - . ID=SmWRKY35;Parent=SmWRKY35.1

SmWRKY35 . exon 72702 73143 . - . ID=exon:SmWRKY35;Parent=SmWRKY35

SmWRKY35 . intron 73144 73880 0.82 - . ID=intron:SmWRKY35;Parent=SmWRKY35

SmWRKY35 . exon 73881 74042 . - . ID=exon:SmWRKY35;Parent=SmWRKY35

SmWRKY35 . intron 74043 74135 1 - . ID=intron:SmWRKY35;Parent=SmWRKY35

SmWRKY35 . exon 74136 74304 . - . ID=exon:SmWRKY35;Parent=SmWRKY35

SmWRKY35 . intron 74305 74391 0.44 - . ID=intron:SmWRKY35;Parent=SmWRKY35

SmWRKY35 . exon 74392 75066 . - . ID=exon:SmWRKY35;Parent=SmWRKY35

SmWRKY35 . exon 81941 81977 . - . ID=exon:SmWRKY35;Parent=SmWRKY35

SmWRKY36 . gene 33229 36457 0.08 - . ID=SmWRKY36.1

SmWRKY36 . mRNA 33229 36457 0.08 - . ID=SmWRKY36;Parent=SmWRKY36.1

SmWRKY36 . exon 33229 34180 . - . ID=exon:SmWRKY36;Parent=SmWRKY36

SmWRKY36 . intron 34181 35180 0.66 - . ID=intron:SmWRKY36;Parent=SmWRKY36

SmWRKY36 . exon 35181 35690 . - . ID=exon:SmWRKY36;Parent=SmWRKY36

SmWRKY36 . intron 35691 36032 1 - . ID=intron:SmWRKY36;Parent=SmWRKY36

SmWRKY36 . exon 36033 36457 . - . ID=exon:SmWRKY36;Parent=SmWRKY36

SmWRKY37 . gene 18086 20821 0.12 + . ID=SmWRKY37.1

SmWRKY37 . mRNA 18086 20821 0.12 + . ID=SmWRKY37;Parent=SmWRKY37.1

SmWRKY37 . exon 18086 18493 . + . ID=exon:SmWRKY37;Parent=SmWRKY37

SmWRKY37 . intron 18494 18672 1 + . ID=intron:SmWRKY37;Parent=SmWRKY37

SmWRKY37 . exon 18673 18819 . + . ID=exon:SmWRKY37;Parent=SmWRKY37

SmWRKY37 . intron 18820 20356 1 + . ID=intron:SmWRKY37;Parent=SmWRKY37

SmWRKY37 . exon 20357 20821 . + . ID=exon:SmWRKY37;Parent=SmWRKY37

SmWRKY38 . gene 13758 15077 0.04 - . ID=SmWRKY38.1

SmWRKY38 . mRNA 13758 15077 0.04 - . ID=SmWRKY38;Parent=SmWRKY38.1

SmWRKY38 . exon 13758 14127 . - . ID=exon:SmWRKY38;Parent=SmWRKY38

SmWRKY38 . intron 14128 14419 0.62 - . ID=intron:SmWRKY38;Parent=SmWRKY38

SmWRKY38 . exon 14420 14536 . - . ID=exon:SmWRKY38;Parent=SmWRKY38

SmWRKY38 . intron 14537 14635 1 - . ID=intron:SmWRKY38;Parent=SmWRKY38

SmWRKY38 . exon 14636 15077 . - . ID=exon:SmWRKY38;Parent=SmWRKY38

SmWRKY39 . gene 5076 6770 0.23 + . ID=SmWRKY39.1

SmWRKY39 . mRNA 5076 6770 0.23 + . ID=SmWRKY39;Parent=SmWRKY39.1

SmWRKY39 . exon 5076 5251 . + . ID=exon:SmWRKY39;Parent=SmWRKY39

SmWRKY39 . intron 5252 5682 0.65 + . ID=intron:SmWRKY39;Parent=SmWRKY39

SmWRKY39 . exon 5683 5832 . + . ID=exon:SmWRKY39;Parent=SmWRKY39

SmWRKY39 . intron 5833 6010 0.96 + . ID=intron:SmWRKY39;Parent=SmWRKY39

SmWRKY39 . exon 6011 6770 . + . ID=exon:SmWRKY39;Parent=SmWRKY39

SmWRKY40 . gene 23972 25957 0.45 - . ID=SmWRKY40.1

SmWRKY40 . mRNA 23972 25957 0.45 - . ID=SmWRKY40;Parent=SmWRKY40.1

SmWRKY40 . exon 23972 24628 . - . ID=exon:SmWRKY40;Parent=SmWRKY40

SmWRKY40 . intron 24629 25111 0.9 - . ID=intron:SmWRKY40;Parent=SmWRKY40

SmWRKY40 . exon 25112 25255 . - . ID=exon:SmWRKY40;Parent=SmWRKY40

SmWRKY40 . intron 25256 25376 1 - . ID=intron:SmWRKY40;Parent=SmWRKY40

SmWRKY40 . exon 25377 25957 . - . ID=exon:SmWRKY40;Parent=SmWRKY40

SmWRKY41 . gene 27856 31379 0.02 + . ID=SmWRKY41.1

SmWRKY41 . mRNA 27856 31379 0.02 + . ID=SmWRKY41;Parent=SmWRKY41.1

SmWRKY41 . exon 27856 28023 . + . ID=exon:SmWRKY41;Parent=SmWRKY41

SmWRKY41 . exon 28441 28938 . + . ID=exon:SmWRKY41;Parent=SmWRKY41

SmWRKY41 . intron 28939 29107 0.58 + . ID=intron:SmWRKY41;Parent=SmWRKY41

SmWRKY41 . exon 29108 29243 . + . ID=exon:SmWRKY41;Parent=SmWRKY41

SmWRKY41 . intron 29244 29934 0.87 + . ID=intron:SmWRKY41;Parent=SmWRKY41

SmWRKY41 . exon 29935 30109 . + . ID=exon:SmWRKY41;Parent=SmWRKY41

SmWRKY41 . intron 30110 30764 0.74 + . ID=intron:SmWRKY41;Parent=SmWRKY41

SmWRKY41 . exon 30765 31379 . + . ID=exon:SmWRKY41;Parent=SmWRKY41

SmWRKY42 . gene 44246 46081 0.42 + . ID=SmWRKY42.1

SmWRKY42 . mRNA 44246 46081 0.42 + . ID=SmWRKY42;Parent=SmWRKY42.1

SmWRKY42 . exon 44246 44609 . + . ID=exon:SmWRKY42;Parent=SmWRKY42

SmWRKY42 . intron 44610 44710 1 + . ID=intron:SmWRKY42;Parent=SmWRKY42

SmWRKY42 . exon 44711 44884 . + . ID=exon:SmWRKY42;Parent=SmWRKY42

SmWRKY42 . intron 44885 45026 0.93 + . ID=intron:SmWRKY42;Parent=SmWRKY42

SmWRKY42 . exon 45027 46081 . + . ID=exon:SmWRKY42;Parent=SmWRKY42

SmWRKY43 . gene 42836 47147 0.19 + . ID=SmWRKY43.1

SmWRKY43 . mRNA 42836 47147 0.19 + . ID=SmWRKY43;Parent=SmWRKY43.1

SmWRKY43 . exon 42836 43318 . + . ID=exon:SmWRKY43;Parent=SmWRKY43

SmWRKY43 . intron 43319 45037 0.99 + . ID=intron:SmWRKY43;Parent=SmWRKY43

SmWRKY43 . exon 45038 45169 . + . ID=exon:SmWRKY43;Parent=SmWRKY43

SmWRKY43 . intron 45170 46351 0.99 + . ID=intron:SmWRKY43;Parent=SmWRKY43

SmWRKY43 . exon 46352 46675 . + . ID=exon:SmWRKY43;Parent=SmWRKY43

SmWRKY43 . intron 46676 46812 1 + . ID=intron:SmWRKY43;Parent=SmWRKY43

SmWRKY43 . exon 46813 47147 . + . ID=exon:SmWRKY43;Parent=SmWRKY43

SmWRKY44 . gene 5896 7992 0.26 + . ID=SmWRKY44.1

SmWRKY44 . mRNA 5896 7992 0.26 + . ID=SmWRKY44;Parent=SmWRKY44.1

SmWRKY44 . exon 5896 6027 . + . ID=exon:SmWRKY44;Parent=SmWRKY44

SmWRKY44 . intron 6028 6124 0.99 + . ID=intron:SmWRKY44;Parent=SmWRKY44

SmWRKY44 . exon 6125 6187 . + . ID=exon:SmWRKY44;Parent=SmWRKY44

SmWRKY44 . intron 6188 6296 1 + . ID=intron:SmWRKY44;Parent=SmWRKY44

SmWRKY44 . exon 6297 6650 . + . ID=exon:SmWRKY44;Parent=SmWRKY44

SmWRKY44 . intron 6651 6736 1 + . ID=intron:SmWRKY44;Parent=SmWRKY44

SmWRKY44 . exon 6737 6853 . + . ID=exon:SmWRKY44;Parent=SmWRKY44

SmWRKY44 . intron 6854 7285 1 + . ID=intron:SmWRKY44;Parent=SmWRKY44

SmWRKY44 . exon 7286 7992 . + . ID=exon:SmWRKY44;Parent=SmWRKY44

SmWRKY45 . gene 16638 22087 0.16 - . ID=SmWRKY45.1

SmWRKY45 . mRNA 16638 22087 0.16 - . ID=SmWRKY45;Parent=SmWRKY45.1

SmWRKY45 . exon 16638 17073 . - . ID=exon:SmWRKY45;Parent=SmWRKY45

SmWRKY45 . intron 17074 20142 0.64 - . ID=intron:SmWRKY45;Parent=SmWRKY45

SmWRKY45 . exon 16638 17073 . - . ID=exon:SmWRKY45;Parent=SmWRKY45

SmWRKY45 . intron 17074 20142 0.64 - . ID=intron:SmWRKY45;Parent=SmWRKY45

SmWRKY45 . exon 20143 20304 . - . ID=exon:SmWRKY45;Parent=SmWRKY45

SmWRKY45 . intron 20305 21272 0.82 - . ID=intron:SmWRKY45;Parent=SmWRKY45

SmWRKY45 . exon 21273 21346 . - . ID=exon:SmWRKY45;Parent=SmWRKY45

SmWRKY45 . intron 21347 21576 0.99 - . ID=intron:SmWRKY45;Parent=SmWRKY45

SmWRKY45 . exon 21577 22087 . - . ID=exon:SmWRKY45;Parent=SmWRKY45

SmWRKY46 . gene 26688 29407 0.14 - . ID=SmWRKY46.1

SmWRKY46 . mRNA 26688 29407 0.14 - . ID=SmWRKY46;Parent=SmWRKY46.1

SmWRKY46 . exon 26688 27161 . - . ID=exon:SmWRKY46;Parent=SmWRKY46

SmWRKY46 . intron 27162 28286 0.97 - . ID=intron:SmWRKY46;Parent=SmWRKY46

SmWRKY46 . exon 28287 28430 . - . ID=exon:SmWRKY46;Parent=SmWRKY46

SmWRKY46 . intron 28431 28967 0.91 - . ID=intron:SmWRKY46;Parent=SmWRKY46

SmWRKY46 . exon 28968 29407 . - . ID=exon:SmWRKY46;Parent=SmWRKY46

SmWRKY47 . gene 15406 28612 0.06 + . ID=SmWRKY47.1

SmWRKY47 . mRNA 15406 28612 0.06 + . ID=SmWRKY47;Parent=SmWRKY47.1

SmWRKY47 . exon 15406 15813 . + . ID=exon:SmWRKY47;Parent=SmWRKY47

SmWRKY47 . intron 15814 16517 0.77 + . ID=intron:SmWRKY47;Parent=SmWRKY47

SmWRKY47 . exon 16518 16670 . + . ID=exon:SmWRKY47;Parent=SmWRKY47

SmWRKY47 . intron 16671 17238 0.92 + . ID=intron:SmWRKY47;Parent=SmWRKY47

SmWRKY47 . exon 17239 17569 . + . ID=exon:SmWRKY47;Parent=SmWRKY47

SmWRKY47 . intron 17570 19433 0.79 + . ID=intron:SmWRKY47;Parent=SmWRKY47

SmWRKY47 . exon 19434 19656 . + . ID=exon:SmWRKY47;Parent=SmWRKY47

SmWRKY47 . intron 19657 19746 1 + . ID=intron:SmWRKY47;Parent=SmWRKY47

SmWRKY47 . exon 19747 19824 . + . ID=exon:SmWRKY47;Parent=SmWRKY47

SmWRKY47 . intron 19825 20246 1 + . ID=intron:SmWRKY47;Parent=SmWRKY47

SmWRKY47 . exon 20247 20377 . + . ID=exon:SmWRKY47;Parent=SmWRKY47

SmWRKY47 . intron 20378 22669 0.66 + . ID=intron:SmWRKY47;Parent=SmWRKY47

SmWRKY47 . exon 22670 22741 . + . ID=exon:SmWRKY47;Parent=SmWRKY47

SmWRKY47 . intron 22742 23119 0.85 + . ID=intron:SmWRKY47;Parent=SmWRKY47

SmWRKY47 . exon 23120 23605 . + . ID=exon:SmWRKY47;Parent=SmWRKY47

SmWRKY47 . intron 23606 23757 0.95 + . ID=intron:SmWRKY47;Parent=SmWRKY47

SmWRKY47 . exon 23758 24525 . + . ID=exon:SmWRKY47;Parent=SmWRKY47

SmWRKY47 . intron 24526 25851 0.69 + . ID=intron:SmWRKY47;Parent=SmWRKY47

SmWRKY47 . exon 25852 26079 . + . ID=exon:SmWRKY47;Parent=SmWRKY47

SmWRKY47 . intron 26080 27931 1 + . ID=intron:SmWRKY47;Parent=SmWRKY47

SmWRKY47 . exon 27932 28612 . + . ID=exon:SmWRKY47;Parent=SmWRKY47

SmWRKY48 . gene 42478 47827 0.38 - . ID=SmWRKY48.1

SmWRKY48 . mRNA 42478 47827 0.38 - . ID=SmWRKY48;Parent=SmWRKY48.1

SmWRKY48 . exon 42478 42974 . - . ID=exon:SmWRKY48;Parent=SmWRKY48

SmWRKY48 . intron 42975 43619 0.97 - . ID=intron:SmWRKY48;Parent=SmWRKY48

SmWRKY48 . exon 43620 43778 . - . ID=exon:SmWRKY48;Parent=SmWRKY48

SmWRKY48 . intron 43779 43874 1 - . ID=intron:SmWRKY48;Parent=SmWRKY48

SmWRKY48 . exon 43875 44578 . - . ID=exon:SmWRKY48;Parent=SmWRKY48

SmWRKY48 . intron 44579 47346 0.88 - . ID=intron:SmWRKY48;Parent=SmWRKY48

SmWRKY48 . exon 47347 47827 . - . ID=exon:SmWRKY48;Parent=SmWRKY48

SmWRKY49 . gene 26256 30719 0.14 + . ID=SmWRKY49.1

SmWRKY49 . mRNA 26256 30719 0.14 + . ID=SmWRKY49;Parent=SmWRKY49.1

SmWRKY49 . exon 26256 26995 . + . ID=exon:SmWRKY49;Parent=SmWRKY49

SmWRKY49 . intron 26996 27594 1 + . ID=intron:SmWRKY49;Parent=SmWRKY49

SmWRKY49 . exon 27595 27918 . + . ID=exon:SmWRKY49;Parent=SmWRKY49

SmWRKY49 . intron 27919 28001 0.48 + . ID=intron:SmWRKY49;Parent=SmWRKY49

SmWRKY49 . exon 28002 28274 . + . ID=exon:SmWRKY49;Parent=SmWRKY49

SmWRKY49 . intron 28275 29647 0.8 + . ID=intron:SmWRKY49;Parent=SmWRKY49

SmWRKY49 . exon 29648 29952 . + . ID=exon:SmWRKY49;Parent=SmWRKY49

SmWRKY49 . intron 29953 30069 1 + . ID=intron:SmWRKY49;Parent=SmWRKY49

SmWRKY49 . exon 30070 30186 . + . ID=exon:SmWRKY49;Parent=SmWRKY49

SmWRKY49 . intron 30187 30358 0.99 + . ID=intron:SmWRKY49;Parent=SmWRKY49

SmWRKY49 . exon 30359 30719 . + . ID=exon:SmWRKY49;Parent=SmWRKY49

SmWRKY50 . gene 25986 29695 0.07 + . ID=SmWRKY50.1

SmWRKY50 . mRNA 25986 29695 0.07 + . ID=SmWRKY50;Parent=SmWRKY50.1

SmWRKY50 . exon 25986 26042 . + . ID=exon:SmWRKY50;Parent=SmWRKY50

SmWRKY50 . exon 26359 26571 . + . ID=exon:SmWRKY50;Parent=SmWRKY50

SmWRKY50 . intron 26572 27712 0.93 + . ID=intron:SmWRKY50;Parent=SmWRKY50

SmWRKY50 . exon 27713 28353 . + . ID=exon:SmWRKY50;Parent=SmWRKY50

SmWRKY50 . intron 28354 28633 1 + . ID=intron:SmWRKY50;Parent=SmWRKY50

SmWRKY50 . exon 28634 28792 . + . ID=exon:SmWRKY50;Parent=SmWRKY50

SmWRKY50 . intron 28793 28887 0.99 + . ID=intron:SmWRKY50;Parent=SmWRKY50

SmWRKY50 . exon 28888 29695 . + . ID=exon:SmWRKY50;Parent=SmWRKY50

SmWRKY51 . gene 4136 6176 0.14 + . ID=SmWRKY51.1

SmWRKY51 . mRNA 4136 6176 0.14 + . ID=SmWRKY51;Parent=SmWRKY51.1

SmWRKY51 . exon 4136 4506 . + . ID=exon:SmWRKY51;Parent=SmWRKY51

SmWRKY51 . intron 4507 4629 0.92 + . ID=intron:SmWRKY51;Parent=SmWRKY51

SmWRKY51 . exon 4630 4755 . + . ID=exon:SmWRKY51;Parent=SmWRKY51

SmWRKY51 . intron 4756 5326 0.92 + . ID=intron:SmWRKY51;Parent=SmWRKY51

SmWRKY51 . exon 5327 6176 . + . ID=exon:SmWRKY51;Parent=SmWRKY51

SmWRKY52 . gene 707 4627 0.29 - . ID=SmWRKY52.1

SmWRKY52 . mRNA 707 4627 0.29 - . ID=SmWRKY52;Parent=SmWRKY52.1

SmWRKY52 . exon 707 1536 . - . ID=exon:SmWRKY52;Parent=SmWRKY52

SmWRKY52 . intron 1537 1613 0.83 - . ID=intron:SmWRKY52;Parent=SmWRKY52

SmWRKY52 . exon 1614 1727 . - . ID=exon:SmWRKY52;Parent=SmWRKY52

SmWRKY52 . intron 1728 1911 0.96 - . ID=intron:SmWRKY52;Parent=SmWRKY52

SmWRKY52 . exon 1912 2415 . - . ID=exon:SmWRKY52;Parent=SmWRKY52

SmWRKY52 . intron 2416 3860 0.97 - . ID=intron:SmWRKY52;Parent=SmWRKY52

SmWRKY52 . exon 3861 3935 . - . ID=exon:SmWRKY52;Parent=SmWRKY52

SmWRKY52 . intron 3936 4339 0.97 - . ID=intron:SmWRKY52;Parent=SmWRKY52

SmWRKY52 . exon 4340 4627 . - . ID=exon:SmWRKY52;Parent=SmWRKY52

SmWRKY53 . gene 1 9954 0.03 + . ID=SmWRKY53.1

SmWRKY53 . mRNA 1 9954 0.03 + . ID=SmWRKY53;Parent=SmWRKY53.1

SmWRKY53 . intron 1 131 0.31 + . ID=intron:SmWRKY53;Parent=SmWRKY53

SmWRKY53 . exon 132 196 . + . ID=exon:SmWRKY53;Parent=SmWRKY53

SmWRKY53 . intron 197 1157 0.2 + . ID=intron:SmWRKY53;Parent=SmWRKY53

SmWRKY53 . exon 1158 1238 . + . ID=exon:SmWRKY53;Parent=SmWRKY53

SmWRKY53 . intron 1239 1921 0.27 + . ID=intron:SmWRKY53;Parent=SmWRKY53

SmWRKY53 . exon 1922 2021 . + . ID=exon:SmWRKY53;Parent=SmWRKY53

SmWRKY53 . intron 2022 8403 0.29 + . ID=intron:SmWRKY53;Parent=SmWRKY53

SmWRKY53 . exon 8404 8675 . + . ID=exon:SmWRKY53;Parent=SmWRKY53

SmWRKY53 . intron 8676 9143 0.99 + . ID=intron:SmWRKY53;Parent=SmWRKY53

SmWRKY53 . exon 9144 9248 . + . ID=exon:SmWRKY53;Parent=SmWRKY53

SmWRKY53 . intron 9249 9355 0.91 + . ID=intron:SmWRKY53;Parent=SmWRKY53

SmWRKY53 . exon 9356 9954 . + . ID=exon:SmWRKY53;Parent=SmWRKY53

SmWRKY54 . gene 1 1077 0.39 - . ID=SmWRKY54.1

SmWRKY54 . mRNA 1 1077 0.39 - . ID=SmWRKY54;Parent=SmWRKY54.1

SmWRKY54 . exon 1 379 . - . ID=exon:SmWRKY54;Parent=SmWRKY54

SmWRKY54 . intron 380 532 1 - . ID=intron:SmWRKY54;Parent=SmWRKY54

SmWRKY54 . exon 533 646 . - . ID=exon:SmWRKY54;Parent=SmWRKY54

SmWRKY54 . intron 647 721 1 - . ID=intron:SmWRKY54;Parent=SmWRKY54

SmWRKY54 . exon 722 1077 . - . ID=exon:SmWRKY54;Parent=SmWRKY54

SmWRKY55 . gene 10884 12109 0.27 - . ID=SmWRKY55.1

SmWRKY55 . mRNA 10884 12109 0.27 - . ID=SmWRKY55;Parent=SmWRKY55.1

SmWRKY55 . exon 10884 11611 . - . ID=exon:SmWRKY55;Parent=SmWRKY55

SmWRKY55 . intron 11612 11764 1 - . ID=intron:SmWRKY55;Parent=SmWRKY55

SmWRKY55 . exon 11765 11878 . - . ID=exon:SmWRKY55;Parent=SmWRKY55

SmWRKY55 . intron 11879 11953 1 - . ID=intron:SmWRKY55;Parent=SmWRKY55

SmWRKY55 . exon 11954 12109 . - . ID=exon:SmWRKY55;Parent=SmWRKY55

SmWRKY56 . gene 5038 7095 0.01 + . ID=SmWRKY56.1

SmWRKY56 . mRNA 5038 7095 0.01 + . ID=SmWRKY56;Parent=SmWRKY56.1

SmWRKY56 . exon 5038 5900 . + . ID=exon:SmWRKY56;Parent=SmWRKY56

SmWRKY56 . intron 5901 6035 0.4 + . ID=intron:SmWRKY56;Parent=SmWRKY56

SmWRKY56 . exon 6036 6161 . + . ID=exon:SmWRKY56;Parent=SmWRKY56

SmWRKY56 . intron 6162 6701 0.44 + . ID=intron:SmWRKY56;Parent=SmWRKY56

SmWRKY56 . exon 6702 7095 . + . ID=exon:SmWRKY56;Parent=SmWRKY56

SmWRKY57 . gene 4303 6057 0.43 - . ID=SmWRKY57.1

SmWRKY57 . mRNA 4303 6057 0.43 - . ID=SmWRKY57;Parent=SmWRKY57.1

SmWRKY57 . exon 4303 5065 . - . ID=exon:SmWRKY57;Parent=SmWRKY57

SmWRKY57 . intron 5066 5158 1 - . ID=intron:SmWRKY57;Parent=SmWRKY57

SmWRKY57 . exon 5159 5272 . - . ID=exon:SmWRKY57;Parent=SmWRKY57

SmWRKY57 . intron 5273 5380 1 - . ID=intron:SmWRKY57;Parent=SmWRKY57

SmWRKY57 . exon 5381 5662 . - . ID=exon:SmWRKY57;Parent=SmWRKY57

SmWRKY57 . intron 5663 5742 0.99 - . ID=intron:SmWRKY57;Parent=SmWRKY57

SmWRKY57 . exon 5743 6057 . - . ID=exon:SmWRKY57;Parent=SmWRKY57

SmWRKY58 . gene 1 5189 0.07 + . ID=SmWRKY58.1

SmWRKY58 . mRNA 1 5189 0.07 + . ID=SmWRKY58;Parent=SmWRKY58.1

SmWRKY58 . intron 1 293 0.31 + . ID=intron:SmWRKY58;Parent=SmWRKY58

SmWRKY58 . exon 294 457 . + . ID=exon:SmWRKY58;Parent=SmWRKY58

SmWRKY58 . intron 458 2020 0.67 + . ID=intron:SmWRKY58;Parent=SmWRKY58

SmWRKY58 . exon 2021 2129 . + . ID=exon:SmWRKY58;Parent=SmWRKY58

SmWRKY58 . intron 2130 4045 0.6 + . ID=intron:SmWRKY58;Parent=SmWRKY58

SmWRKY58 . exon 4046 4216 . + . ID=exon:SmWRKY58;Parent=SmWRKY58

SmWRKY58 . intron 4217 4291 0.75 + . ID=intron:SmWRKY58;Parent=SmWRKY58

SmWRKY58 . exon 4292 4405 . + . ID=exon:SmWRKY58;Parent=SmWRKY58

SmWRKY58 . intron 4406 4558 0.98 + . ID=intron:SmWRKY58;Parent=SmWRKY58

SmWRKY58 . exon 4559 5189 . + . ID=exon:SmWRKY58;Parent=SmWRKY58
