# Supplementary material for: Identification of WRKY gene family and characterization of cold stress-responsive WRKY genes in eggplant
Source: PeerJ. 2020 Mar 17;8:e8777. doi: 10.7717/peerj.8777 (PMC7083166; doi:10.7717/peerj.8777)
Supplement: Data S2 [file peerj-08-8777-s002.doc]

**Data S2 The specific primers for VIGS vector and qRT-PCR**

| Gene Number | VIGS | qRT-PCR |
| --- | --- | --- |
| SmWRKY26 | cggaattccagcagccaggggcagtgg  ggggtaccgcaaagcaatgactccataaac | gccttccgtgacatctcatc  aaacatgtcatcccgaggct |
| SmWRKY32 | cggaattcgtggtagtggcagctacgttatg  ggggtaccgttaaggaaagagctgaagaat | tgaacccgaggcaaagagat  ccggacagccagtaaatgtg |
| Actin |  | gcagctcctccatcgaaaag  ccgatcagcaataccaggg |
