# Supplementary material for: Identification of WRKY gene family and characterization of cold stress-responsive WRKY genes in eggplant
Source: PeerJ. 2020 Mar 17;8:e8777. doi: 10.7717/peerj.8777 (PMC7083166; doi:10.7717/peerj.8777)
Supplement: Data S3 [file peerj-08-8777-s003.doc]

>Sme2.5_00009.1_g00017.1

MDCGFNCDYNSLINELTQGLEYVKQLRASNNIQELDFHMQMILFSFEKSLSILNWNGSVTQTPLLIAPPESSISVEENPRSDDQDFRYVSKKRKQMPTWSEQVRVSAENGYEGPTDDGYSWRKYGQKDILGAKYPRSYYRCTYRLMQNCWATKQVQRSDDDPALFEITYKGTHTCNQTFNCAATQPKSPEKHRFKKQANNPITPPSNQMLANLQANLRVNTNDLHQKEATCSFPFSPTFSGFVDENLHFQISQVDDNLVGGYSPSFVSPTTPESSYFSLPSYQMNDSQRIHNAHHSESDQPDLFSANTSSTSSPIIGLEFPLEQVELDPNFPFDNSEFFR

>Sme2.5_00013.1_g00024.1

MDGVFTWDYYSLINELTQGMEHTKQLRTYLSSVASTSGAIPELLLQKILSSYEQSLIILKWSGSTVQSLPAAGAIESMVSGDGNPRSDEKERSYNDHQKIIHISKKRGKPPFSPCCPNTMCDIIVDIVTILDDGTQILKVFHLKDRLSGKPHFKVCFIPLNTEFALQVFSFDLAQFESFGLQGNVTWFLMGVSRKSQFTAQLPVRTQSGYEGPIDDGYSWIKYGQKCILGAKFPRGYYRCTFFKNCWAKKKIQRTDVDPIVFEITYKGYHTCQYRQVTNSTHQATSLEKQANHPSLIDGSYSPSFVSAEGGFDSLEGPIDGGYSWRKYGEKDILGAEYHRSFYRCKYHNMQNCRATKEVQMSDDDPTAFEIVYKGSHTCHLATNSAQEATSSEKEASHHSLIVGGYSPSFVSPKTPESNHFSVSSRQMNGLGIVHNLDHSESYLSDIGIFCELRHSDSDRTDTFSGNTSTTSSSIGGQFAIVSGARHDFPFWTKRHSETEANTPKKNDKDE

>Sme2.5_00013.1_g00025.1

MDCPSNWEYKTVINELTQGIEHTKQLREHFHSAGSTFENQELLLQKILSSYEQSLLILKWSGSTVQSPQPLPPTCGAIESSVSVDGSPKSDDMKRSFQDHHELINISKKRKSQPTWTEQVKVSAESGFEGPTDDGYSWRKYGQKDILGAKYPRSYYRCTYRHMQNCWATKQVQRSDDDPTVFDITYRGSHNCHHATYSALQPTSPEKPEFKNQAVYQSGQYSNQVLMNLRANLRVNTNDLDKNETAACHFTFPTTFSSSGLTDDNRHFQISHVDENLIGNGYSPSFISPTTPESNYFSISSSSQMNGYGMVHNLHHSESDLTDIFSANTSTTSSPIVGDFSLDHLELDPNFPFDNPRFFS

>Sme2.5_00016.1_g00020.1 /pseudo

MDDEIDSSKSAVPENSNLEQQKNSNKIENSSVHNSDGVINGNNGAENLRKSKTEGLISETLVHPAVQSSVQIETDSQSEFDTEFSPSYQLSEVPVEYELSPFGFSNKIKDRNSVTKSGSLKAQARTEHQRRVPDEASSLELSSISVAQSISSVPSPTLAERRLSTVVNCSTGEVAKQSSDVKVLPLVPVLKTPTRDGYNWRKYGQKQVKSPQGSRSYYRCTHSECFAKKIECSDHTNRVMEIIYRNRHNHDPPQRVNCSRESKSAVLSSPTNGKSIIAHPNQNSNETVASSLKENLQESFPIVETANQDSGESDTDTEITIKEEHSDEPDQTKRQVLS

>Sme2.5_00029.1_g00022.1

MAVELMMDYRNTRNTNCINFVTQLEEKSVVQEAASGLESVEKLIRLLSETQSQQIQQQNKSPMEIEMVADAAVTKFKKVISLLDRNRTGHARFRRAPLAGTTTSPSPSNSSKDFGDTKVYSPTPIQQVPLISHDHFNPLVPKTISFSYSPEMSRTNSFNISSLTGETESKQHSSPNSAFQITNLSSQVSNSAGKPPLSSASLKRKCSFSENALSGKCSGSSGRCHCSKRRKLRLKRVIRVPAISMKLADIPPDDYSWRKYGQKPIKGSPHPRGYYKCSSVRGCPARKHVERALDDPTMLIVTYEGEHNHSLSVAETSSLILESS

>Sme2.5_00038.1_g00016.1 /TE

MAVDLMTTGYRNDSFSSKMEETAVQEAAAAGLQSVEKLIRLLSQSHQHQHQQQQHQKPNFQDSSSSSLTNSSVSADYQAVADAAVNKFKKFISLLDKNRTGHARFRRGPITSRPPPPPPPPPSSLSKPQQNKLQLQQPIKNQNPQIEEIEKSQTTKIYCPTPIQRLPPLPHNHLQIVKNGSIERKEASTTINFASPSPATSFMSSLTGETESLQQSLSSGFQITNLSQVSSAGRPPLSTSSFKRKCSSMDDAALKCSSAGGSSGRCHCPKKRKSRVKRVVRVPAISMKMADIPPDDYSWRKYGQKPIKGSPHPRGYYKCSSVRGCPARKHVERALDDPAMLIVTYEGEHNHSHSITETPAAHVLESS

>Sme2.5_00100.1_g00012.1

MEDTHSQQQQDHHSSSEQLRDGETSSGGLRHNSAGSNGGAKYKLMTPAMLPISRSACITIPPGLSPSSFLESPVLLSNIKAEPSPTTGSFSKFQTVQGSGGAAAFLLTRGYSSGNTYIEGKASCFEFKCAGGSSSTSGSLATEHVITTVLNQQQNEPLKEVQDQSHPQLLVPSSPAKHEMEASKEPSISAPINVDASSKEESLYEPINVDAMNSRGQSNASMQGSHVDHRDVSSVTSERSSDDGYNWRKYGQKLVKGSEFPRSYYKCTYPNCEVKKIFEQSPEGQIREIVYKGSHDHPKPQLSRRFSPGALMSIQEDKCENEACFRGQEVYVEDKFNTNVQTSKTEPSSTPVSPQQADTDGLEGAGLQMHGTSDDMDEDDQFAKRRKMDGGMDITPVIKPIREPRVVVQTVSEVDILDDGYKWRKYGQKVVRGNPNPRSYYKCTNAGCPVRKHVERASHDPKAVITTYEGKHNHDVPAARNNNHEMTGSSPVTGCSRIRPGETNSLSLDLGVGTGYRPDNRNNGQLHTIHSQGVSRSGMMLVQPAAVAARYGIIDSGLSRFGAIDNRVQGPSFETLRLQPSTQSLQNYGKILLGP

>Sme2.5_00161.1_g00022.1

MEERASEVSFPALTIPPRPSLESFFNTSSFSPGPMSLVSSFLAEQSPSFSKLLAEEECSGDPEGSQRPSGYKQNRPVNLELAQSPLFMNPSSLSPSVFLNSPGFLSPLQSPFGMSHQQALAHVTAQAEFSSSYMQMKAEDQCYSQVDSGEELGNVLSTDPKESSWQIKEHLVTGSDNKPLDELGKHFEQPEVSQSENKTSFGTLDKPACDGYNWRKYGQKKVKASECPRSYYKCTQRNCSVKKKVERSIDGHITEITYNGQHNHVQPTKQRKDGSALDNTDFSGVRRDISTHEWTVMNSSDGSSTQMTSELLVERECDEIISNLIDVEERHDEPDAKRTKTAVETVALPHVTIADSKIVLQTRSEVDLLDDGYKWRKYGQKVVKGTQHPRSYYRCTYSGCNVRKQVERASTDPKAVITTYEGKHNHDIPTVVRNRRTSPGPMSLVSSFLAEQSPSFSKLLAEEECSGDPEGSQLTCGNRVPLYFMLSTLQ

>Sme2.5_00196.1_g00004.1

MIFAMSFSSSSLKNSVFVPNGSDQRGHNHHPPGEIHVIFGPMFAGKTTALLRWVNSESKLGRNVVMIKSNKDTRYAVDAVVTHDGTKFPCWSLPDLSSFKHRFGPDAYEKVDVIGIDEAQFFDDLYDFCCNAADIDGKIVIVAGLDGDYLRRKFGSVLDIIPIADTVMKLTARCELCGKRAFFTLRKTDEMQKELIGGANMYMPVCRQHYVHGKSIVESTKKVLESHQVAGCRLGNEDLSGNVRGFHTCERPFSMVKKHSMAKGRKLILINVLPTMGALVLLCAFMSVLLMCDQRRKVKTNVLLDFEYEACVLDFGMAKLLKPDLSNFTVSYKRNQLWGRGNRKLERGIQGEESNTPTRALELFVLHVQIDPNSTTVALLNYSFYSPANYLFENVQHDDWNMILIMGTPKEEATDEVLAENLEQEPDLDPAISKSELKETKSFESISVDVVSGELQKRLSQCDKGINESQSNQEATSLPREQENQSEKAQNLQGLKSEADTSGSSQLSSLPKDSDAKSGGSESSVKGKVVSVLSGKPSDSSDQMQTSNMEIVVSKSDQQRVSCHIKREKALDKLQPRRNPDASVHGLTSDQGTTLFRAPEKPSEDGYNWRKYGQKLVKGNEFTRSYYKCTYPSCLGKKQVERSHDGHITDIHYIGKHEHPETPSVPQMSPELVLPLQMKQPDVPIITALEAEGEKSTMPRETCEPSKPSEAPLALDIVPACGDVKVTPLKRHKLENEVDKDDAPDSKRQKKDTVPTDDTPPIKSHSEPRHIVQTVSEVDIINDGQRWRKYGQKIVKGNPNPRSYYRCSVAGCPVKKHVERASHDPKLVITTYEGHHVHDFPTSRAISQISAAPDSGTTNVREDSRIESGENKHVRESKTEAAENKHVGESKSESGESKHIGESKTEAGENKHVKESRVKSAENKHVEDSRVKSAENKHVGESRNESGGNKHVEESKPESVGNKHVGESIPESGENKHVGLDMAVHIGAN

>Sme2.5_00232.1_g00014.1

MAENENDWDLWAVVRSCSNMNNSVHDDNNVNSTSVLDDHGFHKDPTHENSVTNTQNTTLFPEESDCVGDFTDWFPIENKHYFGLDEVISLSKNLNTNSGIEQVVVEHEEEEEKKKKKVRYSMQSSSTGPGEAFPYRGKSTERYEILAEKLSETDQWRWRKYGKKPTGGSTFLKSYFRCNEVDDCPAKRHVQKSSTDPNKVIVTYRNQHNHPPPNQHIGMVHRSPNAAAPAEDPSFPSST

>Sme2.5_00232.1_g00015.1

MVISMLVIPRFFGNGDINVNNTSVLGNGDINVNNTSVHGNGDINVNNTSVHGNGDINVNNTSVHGNGDINVNNTSGHGNGDINVNTSVLGDQTVHEDPTQGTSVNGDINVDNTSVLDNHSVHEDPTHGNSVNGDINVNNTSVLDNHSVHEDPTHGNSVNTTQDTAHFPEESHSVGTRIPIQELNPATKKTKREIVAEADQWRWKKYEIGHYSTFTGIYYTCGEVENCPAKRLVQKSIRDPNKGIVTCRGQHNHPPLNQHIAMVQRRPNAAVPMEDPSFPSSTSTL

>Sme2.5_00264.1_g00017.1

MNENFALQEAASAGLKSMEHLIRLVSHEPVQVDCREITDFTVSKFKKVISMLDRTGHARFRRGPVQAQAQAQVQAHPDSFTSLSLASSMNFAPAKEAPAMPVQTALTLDFTKPNVDRLIGNSDAVTVAVKSKLMATPTPTNSSSFVSSITGEGSVSNGKQGTSSLLLFPVQAGKVPVTGKRCREHEQSDSISGSKSTCSGKCHCKKRKFRPKKVIRIPSISSRIADIPADEFSWRKYGQKPIKGSAYPRGYYKCSIFRGCPARKHVERAMDDPTMLIVTYEGEHCHTQVAMQENNSQMVNFGKKKE

>Sme2.5_00281.1_g00010.1

MENNKSENDNEMEIDLSLKLDAGEEENEENQIGESSQLNEKTQEKDQELPKNEELSILEKEMKRMKEENKVLRKAVEQTMKDYHDLRAKFSIHQNNHKDHKNFLSLSGNDDTSEGQTRVPKLLDITNANTISSPTSLEDDSMDGDQLGLSLTLVSSNSTTSNKLVEVLEEEQKKDHTTFTHQIQNKPQNLGGLTNHVTTASPPNRKSRVSVRARCESATMNDGCQWRKYGQKIAKGSPNCPRAYYRCTAAPGCPVRKQVQRCLEDMSILITTYEGTHNHPLPVGATAMASTASAAASFMLMDSSISPLLNHNSTLNQFPNYHNNNNLAPNYHHSSSSPYNISNLRNNILNSSDHISSQGNIVLDLTKNQFPFASSSSNTHEIGHSNWMPKLPNYEGNSLYNHHNNNNNNNNNIPPMLAHENMSAIASDPKFRVAVAAAISSLINKDQSHSTGES

>Sme2.5_00386.1_g00007.1

MENKNKADHDDEVCADQENIVHKEATAVNVMVKRERSPPENNSMASSSTHKEQDDQIELAKAEMREVMEENQRLRMHILFLGRTTSDMKKDELSKIVKKDKGHDNEGGVNKTLDLGLDCKFKTIPTECSPVNLSPENSLEDQANKDENGETSTTWPPNKNLKTMRNNGDGDGDGDDVSQQNPTKRARVSVRVRCDATTMNDGCQWRKYGQKIAKGNPCPRAYYRCTVAPNCPVRKQVQRCPEDMSILITTYEGTHNHTLPLSATAMASTTSAAASMLLSGLSSSSDPNPQATATTTTNTNTNNATSANINGLNFYLSDASRHKSSYYFPNSSFSASAPNSHPTITLDLTSTSSSSSSSLSHLNRMSHNFPPRYNYNNNNSSTNLNFSSVLESNSLPISWTNSSYPNQAYNKNNQNFGSLTFSSRPNHENIFQSYLQKNNNISTQSSLPPETIAAATKAITSDPNFHSALAAALTSIIGNTGIENKLNVTEPFPILSSLPSTSNPNKCSSSFSNKPTSSASANNTQQPGNSNLVFFAQSSSSLPFSTSNKGKSTSPSDSKVD

>Sme2.5_00423.1_g00013.1

MEDRLYKSPFFHKQEDSTGTPPDNAADSCFSGDEAAEISMPSPRKRRGAKKKVISVPIIEADGSRSKGEVYPPQDSWSWRKYGQKPIKGSPYPRGYYRCSSSKGCPARKQVERSRLDPTMLLITYCSDHNHQIPAAAATKHHHHNHPTIATTPSNSSPTTSTGTAEDNNAAAAAVTDAAVQDKSSPEEPDPFAYQNDNGFSELAGELGWFSYMGTTTFMESTSTSAVGSTWNDSDVALMLPIREEDQSLFGDLGELPECSVVFRRYGVETPCCGGTG

>Sme2.5_00556.1_g00018.1

MLDEELIRMREENKKLVTMLTNLCENYTSLKTQIIELQQKYSTHEEDNSKLCFSRKRKAEEDCSENYVEEASPKRPREITTDVSTVCVKTNPSDQNSVVKDGYNWRKYGQKVTRDNPSPRAYYKCSFAPTCPVKKKVQRSVNDPSILVATYEGEHNHPQPSQAELTVPLVNQDVTTNPTFLNKFMEDINTSSLQQHLVEQMASSLSKNPSFAATVATAISGLLF

>Sme2.5_00556.1_g00019.1

MDTNLGDKTFSIDLNTNPSLHNTNGSPHETLDEELMRMREENKRLVTMLTTLCEKYTSLQTHLIELLQKYSTHNEEENSKLLSSRKRKAEEECCVNNSDINFEEASPKRPREITTNISTVCVKTTPSDQTSMVKDGYNWRKYGQKVTRDNPFPRAYYKCSFAPTCPVKKKVQRSIEDPSILVAVYEGEHNHPHPCHAEITVPLMNQDVPTDPRFMEDIDTNSIQQHLVEQMASSLTSNPSFTAAVAAAISGKIFEYDLPFK

>Sme2.5_00574.1_g00007.1

MAANNPSASMFDGGFTPILDSPDSDDFSNNLINFELSDIFEIDNWPLQQDPTLIPHYSNYAANQVVNHGEPSNNIGSSSDKKEVKDKVAFRTLSQIEILDDGYKWRKYGKKMVKNSPNPRNYYRCSMEGCPVKKRVERDKEDSRYVITTYEGVHNHPRFDEEEISSKLLRL

>Sme2.5_01030.1_g00008.1

MEGNGYPIDEWNCQLGENFSNNNKRSIAQRCGFNASSISTPATLPPPERETFLTIPPGLTPAALLDSPVMLPISQAPQSPTTGSFQPFSIMINQEQYSLPSSANTQPQSRYNFEFPTASPVDSDTPNSQQNMSGMKLVNCCLNPNINSSNGSDDGYTWRKYGQKTVKGSEFPRSYYKCTHQNCLVKKKVERSLDGHITEIIYKGEHNHHKSRATMSSNSYHLENTNSSTQFEGNKDVRLVSNEVSNSLMSNHQTANTNVLVSAETTPEPSSTLASCDDEDEDRSTSFGDDNDNEFDHKRRRSLSSRTTREPRVVVQIESEIDIVDDGYRWRKYGQKVVKGNPNPRSYYKCTCAGCPVRKHVERAPDNLKSVMTTYEGKHNHQVPSANKKNASHLGTQVEEFEPSFLENDLGYLRFPPNPLFVSPTQISTSYPFTLHKPHFSLPLNFPLGGFHCNHLLLTPNSVSSQSSLAIRDQNHIYNNNTNTNA

>Sme2.5_01060.1_g00010.1

MEEIEEGNKAAVEHCHRVIDLLSSRKHDQNSYTNIVRETGEAVNKFKKVVTLLNSTLGHARVRKSNEFKTPLPRDILVENLNSKNDDQAKALKGLLPFNSPENRVLEVGTNVKCNLSLGSPSLELSSNSRNPLNLGQQTPLPSYNYLQQQQQQRRFLPHPQQPQMKQPAEMLYRQSNSGISLNFDSSTCAPTMSSTRSFISSLSVDGSVANGSNFHLIGASNSADKSLFQNKRKCSERGDEGSMKCGSSGKCHCSKKRKHKVKRSIKIPAISNKLADIPSDEYSWRKYGQKPIKGSPHPRGYYKCSSMRGCPARKHVERCLEDASMLIVTYEGEHNHPRVPSQLDKTSILGDEELCHRIRDFPDCCRFRFVTMLRKFIFLSSCPPSLLLGGMGLNPIPYSTS

>Sme2.5_01071.1_g00003.1

MEDDWDLHAVVRSCTTTTSSAISFNNFPSTTHNNDDSLFSFQHLVDKPRILLNTNTTELRKPFITHQTLLSPISVLPPLQDLPAPSQQQQQQHDVHHLSNMKLIQHKKPLTCTLHPQTPRNKRRKNQLKKVCQVAADGLSSDMWSWRKYGQKPIKGSPYPRGYYKCSTSKGCLARKQVERNRSDPNMFIVTYTGEHNHPMPTHRNSSGIARQKTTKPTTSSPAPENEEISRDEKEDFFEDDDDEFGLDKMEPDDDFFDGLDELKIPATGDSLPEKFPPTLQLPWLVNNAATTAAGGS

>Sme2.5_01077.1_g00010.1

MEGVSFWGTICLNYFGFGLMQRLLLLGACTSVCNSRSSEAEVFFFKVLFHIKVTINVLQVQPSPTTGKFPFSSGMESRNSALMMEDLDKRKENALESMNSSSFSFKPVPETAPSLFPGATSRVNPYNFSQQGFPNIEVSVHSQNSLLSHHVEATQNPTQNGTLHQASDFPRFSAEKDVTSSNVAPESRTFQAVGSNVEHSPPLNELQDEDIDQRGGGDQNVVGAPADDGYNWRKYGQKQVKGSEYPRSYYKCTHPNCPVKKKVERSQEGHITEIIYKGAHNHPKPPPNRRSALGSTNSLGDLQVDGAEQGASGDLGRANIQKAPGAGGGFDWRNNNLEATSSVNMGSEYCNRSASFPAQNNTQLESGDAVDVSSTFSNDEDEDDRATHGSVSQGYDGEGDESESKRRKVETYSADMTGATRAIREPRVVVQTTSEVDILDDGYRWRKYGQKVVKGNPNPRSYYKCTSAGCNVRKHVERASHDLKSVITTYEGKHNHDVPAARNSSHVNSGASNTHPTPVPAPAQNHLHRPEPAQLQNAMARFDRQPSLGSFGMSGRPQLGPTPGFSYGMNQQGGLSSLAMAGFHPHQNKPGEVPIHSYLGQSRPMHDMGFMFPKEEPKVEPMSDPGLNLSNGSSAYQQFMNRLPLGPQM

>Sme2.5_01130.1_g00003.1

MDSTTAIINKDKEEEELLASTKKEDEDQLNSTKCEIFEAKEENERLKCCKFTQENSFDEEAKKENIKNIEKSIFVQNYPPSKMDYYNYDKDFLEQIPQKKARVSVRAVCGTTMMNDGCQWRKYGQKIAKGNPCPRAYYRCTISPSCPVRKQVQRCCDDTSILITTYEGTHNHPLPFSATAMASTTNAAASMLRCTSTSSTSQQFLPNADQNLHGKFNFTNNNLLLATNNNAPTFSLPKTSISTSQSHPTITLDFTTNSSITNSNPSSFNSYTTLCNNFKSQIGASSYLGRPCFSNHFQDQSGYTTVQYSDGVRISDEWIQKNAKVSHLGFDHVT

>Sme2.5_01183.1_g00010.1

MAVDLLGYSNMKEQLDLQEAASAGLKSMDHLIRFVSHQQQMNQTVQPDCREITDYTVSNFRKVISILNRTGHARFRRSSVQVSDSCTALSLSPSTTPAEDTLPPAPVTASVEKEKEKEKYQSKVLTLDFTKPKVAKSTGCEAVPVASSTTSSSFMSTITGEGSVSNGKQFSSMSLPPRPPVSTGKPPIAGKRCRDHDLSDELSGRTSSSGKCHCKKRKSRVKNVIRVPAISSKTADIPADEYSWRKYGQKPIKGSPFPRGYYRCSTVRGCPARKHVERATDDPGMLIVTYGGEHRHVQSTIPGIGAVSGVGAGSFGERMMVFESTGQKNGERLGLQN

>Sme2.5_01372.1_g00013.1 /pseudo

MEFTSLVDTSLDLSFRPAPVLDKVPKQEVGSDFIGLKRENFGVKNDVGDLLEELKRVSSENKKLTEMLTVVCENYNALRKQMMEYMSTQNGVADDSAGSRKRKAESISNPINNNNNNNNNMDVVHESSSSDEESCCKKLREEHIKAKVTIVSVKTDASDTSLIVKDGYQWRKYGQKVTRDNPCPRAYFRCSFAPTCPVKKKVQRSIEDQSIVVATYEGEHNHPQTSKPESGASSYTSTGSRLNVATIAGTTASVPCSTTLNASGPTITLDLTAPNTAGKRDMKMTHSTSSPTGINSFRTSATTTGGEYHNRPEFQQFLIEQMATSLTKDPSFKAALAAAISGKILQHNNQTGR

>Sme2.5_01585.1_g00006.1

MAASSFSFPTSSSFMNTSFTDLLASDDYPTKGLADRIAERTGSGVPKFKSLPPPSLPLSPPPFSPSSYFAIPPGLSPTELLDSPVLLSSSNLLPSPTTGTFPAQAFNWKSSSHQHVKQEDKNCSDFSFQTQVGTAASISQSQTNHVSLGQQGWNYREPAKQNVLSSDQNANGSEYNTLPSFMQNNNNQNNSGNQYNQCIREQKRSDDGYNWRKYGQKQVKGSENPRSYYKCTYPNCPTKKKVERSLDGQITEIVYKGNHNHPKPQSTRRSSSSTASSAIQSYNTQTNEIPDHQSYGSQMDSVATPENSSISFGDDDHEHTSQKSSRSRGDDLDEEEPDSKRWKRESESEGLSVLGGSRTVREPRVVVQTTSDIDILDDGYRWRKYGQKVVKGNPNPRSYYKCTSTGCPVRKHVERASQDIRSVITTYEGKHNHDVPAARGSGNHSINRPVVPTIRPSVTSHQSNYQVPLQSIRPQQSEMGAPFTLEMLQKPNDYGFSGYANSEDSYGNQVQDNNVFSRAKNEPRDDMFMESLLC

>Sme2.5_01670.1_g00011.1

MENNYLGDLADIFRGGSNSTSGEPSTAVPEEWQFPYSAAPMAGQDFGDPFCNLRDPLFHDVDMMQPSSNLEQNDPALFGDSPSIKRRGNMFSRKLDVPCNNTLLPNHDACLLENSALHISSPRNTPIKRRKSLAKKVVCIPAAAPANSRQGGEVVPSDLWAWRKYGQKPIKGSPYPRGYYRCSSSKGCSARKQVERSRTDPNMLVITYTSEHNHPWPTQRNALAGSTRSQTNNSKHTNNIVIPNNPQSQTKEDGGHNDNNNVEQVNPKVKEEVGEEDGHQQEVKNEFSKDSIYQPILPDSSNQCHEDFFADLIELEADPLNLLFSKTLSGEINQVGQKKGIDSFDLYDWSKDT

>Sme2.5_01689.1_g00004.1

MEEDWDLHAVVRGCAASSTAAATSTTTTTSTAAATNSCCNFQPRQDGNFFNFQDPFVPRFDNPTTDFEELHNLYKPFFPKSQQIPLSPQNNNNNIPISPLSVLGGLQDLSPQQTLKQQQHIHQLNSTRPTQPKQSPLKNQLKKVCQVPAEGLSSDMWSWRKYGQKPIKGSPYPRGYYRCSTSKGCLARKQVERNRSDPNMFIVTYTAEHNHPMPTHRNSLAGSTRQKPANSEAGTVSDSNKPSSSSPVSSPATEKQESSREEKEDIFEDDDDEFGSSNMGLDNMEPADDDFFEGLDELAAQATGDCFSDNFPGSMQLTWLSNNATTTAAGGV

>Sme2.5_02107.1_g00005.1

MEDSQSQCHSHSHSHSHQYSQSSPLSSINESSEQVKFASSDAAFSHNVSSAVGSNTGAKYKLMSPAKLPISRSSACITIPPGLSPSSFLESPVLLSNIKAEPSPTTGSFAKFQLMQGSSGSAAFSLMRSSSCGNVYGETTDEFEFKFAIGSSPTSGSLTKEAAVIAPGFNQPQSEPLIQVQKRYLSQSSAPSALVESEMPNSKELSLPAPGSLDVSSIITSAAAADNEEINQRGQSNPSLQGSHVDNKDVSSVTAERSSEDGYNWRKYGQKLVKGSEFPRSYYKCTYPNCEVKKIFERSPDGQITEIVYKGSHDHPKPQPSRRFTAGALTSIQEDRGERDACLTGQEDKFNTDAQTSNTEPSGTPLSPRQADDDGLEGTVSQLHSANDQMDEDDPFAKRRKMDGGMDITPVVKPIREPRVVVQTVSEVDILDDGYRWRKYGQKVVRGNPNPRSYYKCTNAGCPVRKHVERASHDPKAVITTYEGKHNHDVPTARTNNHEMAGSVPVTGGSRVRTEENGSISLDLGVGIGYGMENRRNGQLHTLPAETVCNQVQVSSSSMMVVQPAAVAACYNIVNGGISRFGTIENHVQGTGFETLPLQSSAQCAQNYGRILLGP

>Sme2.5_02381.1_g00007.1

MDKGWGLTLESSSSDRVGTFFKNKPLFGFNLSPRFNAAEMFPVSDEKRAIVNEVDFFSEKKIVVKKENSQPDNSIRADEQFVVNTGLQLVTANAGSDQSTVDDGISSELLLEDKRAKIQLAHLQVELERMNSENQRLKGMLTQVSNSYSALQMHLVTLMQQQQQQQLISRTENTHSHEVVGAKTDEEKQQENNGTIVPRQFMELGPSSSKVDPMDEPTNSHSSSEERTLSGSPRHNIELPPRDKTIGREESPESESWAPNKTPKLMNSSPKPVEQSTEATMRKARVSISDGCQWRKYGQKMAKGNPCPRAYYRCTMAVGCPVRKQVQRCAEDRSILITTYEGTHNHPLPPAAMAMASTTSAAANMLLSGSMPSADGLMSTNFLARAMLPFSSNMATISASAPFPTVTLDLTAQNPNAAMPNYHQRINQPNAPFQFPSPAGLNHPNFVNSMSAPQMPQVLGQHLYNQSKFSGLQVSQDNIHHPSISHDTLSAATAALTADPNFTAALAAAISSIIGGSHPNNGNSPMSGPSSNNN

>Sme2.5_02389.1_g00002.1

MYNSTSSSSQMHTSENHNNFSVKKKGDNKKMKKPRFAFQTRSQVDILDDGYRWRKYGQKAVKNNNYPRSYYRCTHEGCNVKKQVQRLSKDEGVVVTTYEGMHTHPIDKPNDNFEQILHQMHIFPQSSPFVNSQI

>Sme2.5_02587.1_g00015.1

MASSGGNMNTLMNSFSSSQFITSSFSDFLSDNNKNWGFNDERIMNKDEIPKFKSFPPSSLPMISSSSPASPSSYLAFPHSLSPSMLLDSPVLFNNSNTLPSPTTGSFGNLNSKENDSRNSDFSFQSRPATSSSMFHSSAPTNSLEDLMTRQQQATEFATGVKSEVAPIQSFSQENMQNNPAPMHYCQPSQYVREQKAEDGYNWRKYGQKQVKGSENPRSYYKCTFPNCPTKKKVERNLDGHITEIVYKGSHNHPKPQSTRRSSSQSIQNLAYSNLDITNQSNTFLENAQRDSFAVADNSSASFGDEDVDPGSPISKSGENDENEPEAKRWKGDNENEVISSASRTVREPRIVVQTTSDIDILDDGYRWRKYGQKVVKGNPNPRSYYKCTFTGCPVRKHVERASHDLRAVITTYEGKHNHDVPAARGSGSYVMNKPPSGSNTNNMPVVPRPSVLANHSNQGMNFNDSFFNTTQVQPPITLQMLQSSGSSSYSGFGTSTGSYMNQMQPTNNSKPISKEEPKDDLFFSSFLN

>Sme2.5_02680.1_g00006.1

MEVNEAAKIAIARPVASRPRCPIYKSFSELLAGAIDISSTNVHSEMVTAIRPKTVRLKPATNHALVGEPSSQVGVSEAPVGCRSDNILQSVDKPIAKLAPRKSTPLLENKGSSVSDQRQEIAEANAHVQSANEVKQQHDLKTESKRSLLAKSVQEKKTVQSTIASESTEEVPQSLINTSNVDRPSYDGYNWRKYGQKQVKGSEYPRSYYKCTHLKCPVKKKVEKSYDGQIAEIVYRGEHNHPKPQPPKRNLSEGHGRATLGNASKETNNPAWGNQHPQTSEGYICRIDSQNGVRLTVHSAHSSKAPCFYDPSAAAGMHPAVGNSEDATSDEPKTKRRKIKGLSNRAGTSGESTTPYIPNQSTTDSEITEDGFRWRKYGQKVVKGSSYPRSYYRCTSPKCSVRKFVERTMDDPKAYITTYEGKHNHEVPNRRPNSEASKTSSKSSSMKEKS

>Sme2.5_02752.1_g00007.1

MEKVKAWDKETLIAELTQGKEFVNRLKNQFDPLASPEECDLLLEKILSSLDKSLSILNWKGFNETKDPLSSCPSIVDLGYLRDQGQNKKRKKMQQWSKQVRICGTGVDSFNHDDGYSWRKYGQKDILGAIHPRAYYRCTHRDTQGCLATKQVQKSKEDPLVFEVTYKGMHSCKTSQPSIFISYENQKPNQCQIKKQKVEKLNTIKEETGPFTPPVCQSQNTQFFANSIEPSTSESMYLSLFPTHEEEFEIGKLLHLSSTPTSVIDLPFYAHSDLSMDGQLGIHDPDLMIDISEYFE

>Sme2.5_02954.1_g00006.1

MGISMDDVPQSNQEDSPLSNVPEKDTEKVHQKKGPESEGGASESGRVPVLPKKEPYIKSSKSDSPVKDGNSSLVPGKASDFPQQIQIQKMEEVISQSHQEPVTSSTGENALNKLQPRRDPDTSIQDSSSDHAEKTSEDGYNWRKYGQKLVRGNEFTRSYYKCTHPICRAKKQVERSHDGHITNIHYIAKHEHPKPLNSPQISPELVKPSEMRRPDMPTGKPREGEKSTALGEACQSIEPSESLISAAVVSAGGSVPDTVLKPLKSGDEGDNNGSRNLKRRKKEVPTSDDMSPPPKSPSEPRHIVETRSEVDIINDGYRWRKYGQKFVKGNPNPRSYYRCSSSGCPAKKHVERASHDPKLVITAYEGQHDHDIPPSRTVMQNSAEAESTS

>Sme2.5_03205.1_g00005.1

MEKISNNSILGATEMQNNHEEEDIKKAIKDDEFKSARAQVNEVKLENARLKLLLQQIEKDYTCLQARFFNISQSDLNKSTSPTCTIEEDESELVSLRLGRSPSPSEPKKVDKKRSMREDSDDQSNDGLKLGLDYNTGVSESDPIKPSNEPSPGPTSEAAKKVKIDDDQSVKKGAGDDEVSQPNVKRARVSVRTKCDYPTINDGCQWRKYGQKISRGNPCPRSYYRCSVAPSCPVRKQVQRCLEDMSILITTYEGTHNHSLPIEATAMASTTSAAVSMLLSGSSTSSQISKSISKTTPLYLSNSSSNPLPTITLDFTTFPTTSSFTSFNFPSNFQSGSGFLPNSLNFSSPQSSTMPKILGSGCQNYDSTSTLPYHNNLTNIGSSQKQFDVNSNKLKEGSSQQALTETLTKAIASDPSFQSVVAAAISSMVGATKT

>Sme2.5_03353.1_g00002.1

MEDNPVYSFDNYNNHSFTGLISDYNINSSSLGFMELLGFQDWCSSASVFELPKEENYCPAVCVPEEEVKPSAAPVDAGNVLNTPSTPNGSSISSEGHTHTTDGEVENHDQPNTKSKQQLKAKKTICQKKQKEPRFAFMTKSEVDFLEDGYRWRKYGQKAVKNSPFPRNYYRCTNATCSVKKRVERCFSDPTIVMTTYEGKHSHLSPMNTMMPRPSCYPVTPLLPSPLPMQFNFNQSSNNLTNPNLVMNNQLEHAAFVAQGRRFCSTTEMMGDQGLDLQDLMLKQDYNR

>Sme2.5_03471.1_g00002.1

MSNCTASGMSRKRKAENVGLFDARFHGINSELSDEDLSCNKARLVHHDHFQPKKINYTRYVRTTQASHDTALIVKDGYEWRKYGQKVTRDNPYPRAFFKCSFAPTCPVKKKVQRCVDDKSIIVATYEGEHNHSKELLETGEGSSDQLVNGNRRISTSITAVGHSHNKQPEFHKLLKTKWLHP

>Sme2.5_03533.1_g00001.1

MDGRFNNFFVSEQEDSENSPENSSDSPQSGMFNDNKLMTSTSSPKRSRRSIEKRVVSVPIKEVEGSKMKGEISMPPSDSWAWRKYGQKPIKGSPYPRGYYRCSSSKGCPARKQVERSRADPNMLVVTYSCEHNHPWPASRNNQQNHRTTTTTSCTNNNAKTKMKTIASLTAATTIMTSDVAVSEEKPTGNLARPSEPNSDEKFANLGESSSLINANEFGWFSDLIECTSTTMLESPILTEVEVTDIDMSSTLTMREEDVSLFADLGELPECSTVFGRGMMERDEERRRHSLAHWCGTTG

>Sme2.5_03980.1_g00003.1

MGDELRDLYYHQPFQEDSSSSMAQNIHMLDPSFMSYTSEYLHGSSSDNYITNSLGKPFGFSPPSPFCSTKDDQDIIKQDHVDANINYISNDVVGGGSETPVTPNSSISNSSSNGDHEDSNKKDKQVKDESLEDGEDASKKESKVKKKGEKKQRPPKFAFMTKSEVDHLEDGYRWRKYGQKAVKNSPYPRSYYRCTSQKCQVKKRVERSYQDPSIVITTYEGQHNHHLPATLRGSVARMLNPSMLAQPSPLMAPQATFHQEFIMSQMPQFYGHGNAFGNSPMYHQNLNHQQQMQLPTHHHHDYGLLQDMVPSMFNLKQEP

>Sme2.5_03997.1_g00004.1

MGEYDHFANFGDCIPPSPRPTSKALFSSLVANNFEGWPPHMEETNESITRNVVVEPQEHVTWCNSDGKDGVGTGATTDQIVKSSAPSEQATSSRGGLMERMAARAGFNAPRLNTAGLRPPAMSQNQEVNSPYLSIAAGISLSVLLYSPVFLAPISQYFVVVTALVSVFSFSKCLEMLYFKPRIYQKQPLYLDGVGVSTLLALEFIGLRGWGAGSRGPYKWYQSLGLEGRCDGPTCADQWASSIILLVSEPRFEGQIDEDSDGDASVEDGYNWRKYGQNQVKGSEFPRSYYKCAHPNCFVKKEIARNHKGHVTEVIYNGAHNHPKPLPNRISVLRSSNSSSVMQLDNVDPTGTGLNNELALKTIQQGPTAGGLEGKNDSFDTTSLADLHSEYCRGSTTLHSSAAQLG

>Sme2.5_03997.1_g00007.1

MQLDNVDPTGTRLNNQLALKTIQQGPTAGGLEGKNDSFETTSLANLHAEYCRGSTTLHSSTAQLGSADANDISSTFPNNEVDHGTRGNVSLDCNDEGDESESKIRRIERSSTDTSGTRRSIRERSIREPRIVVQTTSEVDLLDDGYRWRKYGQKVVKGNPNPRSYYNCTNPGCNVRKHVERAPFKPTSVITTYDGKQNHNVPLIYCQTSVITTYDGKQNHNVPAERSSNQANSDASNSRSKPRTTNAQGHAGRTEPTQVQNSRKRYGRAPSFDPTSQFNSSGTNQQQGLTGHAMTGFNSDQRQFSAPADSSGTNQQQGLTGHAMTGFNSDQRQFSAPADSSGTNQQQGLTGHAMTGFNSDQRQFSAPAHSSGTKQQQSLTGHAMTGFNSDQRQFSAPADSSGTNQQQSLTGHAMTRFSSHQHQFSAPAHTYPGRPQPVNDAGFMLPER

>Sme2.5_04027.1_g00002.1

MEETIATILYGCKLGREVEANLPNWGNQRGVLLSKTEEIIGVFNNVRERLISQQQQQQQQAVQEWLSSSGQLFHAADAATTAYAVHGLPQENDQKGGGGSSTMVPMDASHDSSRAASSSSQRQRTRKGDTDRRIVRVSAPRMGNLELPPEDGFTWRKYGQKEILGSRFPRAYYRCTHQKLYHCPAKKQVQRLDDDPYVFEVTYRSQHICYMSATAPTVPLPLVEEITHQTTTTPPPAPLLLPPPTSASLSGHWLSMDIKPQIDQAGTSYTTTPFDIQRDFGHGGSVGSLASICNVVTAGSGGGAGPSGSRFGREVDYQPVVDMADAMFNSGSSSNTSMDIIFSSMDDKWDTAEKKR

>Sme2.5_04190.1_g00001.1

MEFTSLVDTSLDLNFKPLRVVDEAPKQEVESNFIGLGRDVVPAKDEAGDLMEELNRLSAENKKLTEMLTIMCQNYNSLRNQLTEYLSKQNSSGSGSDHGQNSDGSKNRKVVNNNNEIVKSSVQVLNSESSSSDEDASTKKPREEHIKTKTSRVYMRTEASDTSLIVKDGYQWRKYGQKVTRDNPSPRAYFKCSFAPTCPVKKKVQRSVEDQSILVATYEGEHNHCKVDNSGPIATTSPSSRFNPKNNVPSGPGPTFTLDLSEPKTFPQKDPKRQVNSNTSTSNASGQKRKSPASDQQQQNRPELQHFLIEQMASSLTKDPSFQAALAAAISGKFLQNNK

>Sme2.5_04253.1_g00002.1

MERGEERNQLNNYNLQVSFSSSSPANNIHELGFVHFADHNLSFLAPSSQSSQNLQAAAASVSVTPPAAINTTGGGIGFSHNELLVNRPSWSNNDQVETLDPKAINDENCSGNASEGNNSWWKSSSSDKGKVKIRRKLREPRFCFQTRSDIDVLDDGYKWRKYGQKVVKNSLHPRSYYRCTHSNCRVKKRVERLSEDCRMVITTYEGRHNHTPSDDSNSSDQDCFTSF

>Sme2.5_04516.1_g00005.1

MEKENNVKREMITPIGNGNGNGNEDENSSTFLMNNPSFDIEKDYSLGFLLETMFGTIDDYTTSTTTSSIFDLLMMPLPPHHHQPPTPPAPSPSPREIITCSSVVPESSTISSPSIELVADDYKKDGEQDHEDKYNKKLKPNKKQQKGKREPRFAFMTKSEVDHLDDGFRWRKYGQKAVKNAPFPRSYYRCTTTLCGVKKRVERSIQDPSIVITTYEGTHTHSCPMTPRGYIGVQAVTTTYGGARVGCGDSGGRTYNYGDSSYNSLQERRFWNSSNSSLAKDDHGLLQDMVSSRMKRDLIEE

>Sme2.5_04517.1_g00003.1 /TE

MLMEKYSWSYEDELIKELLDDESPFFLAPQHDSTSSKSSISSHDDIESGLSITSNGVLQSHDARNLGLDMMMSKQEAHEAKYTLRIKTCGNAMADDGYKWRKYGQKSIKNSPFPRSYYKCTNPRCGAKKQVEKSSDEPNTFIITYEGLHLHFTYPFITLDPSQLLDQPNKKPKLTHSKAQNTSEVDESPKFVNPGPLGFGEMGSQELDFGGMGSQGLLEDMVPLMGVGYGVRSAVVDEVAARGACITRVDGGLGFARTYLTLIGGGRQGLSKAYLSELDSVFASPKLRRFFCSQGPKRRNYENYYPKNKKEIPKANNNQKAESGKEEGSSEQGNPQENFIKLNYNLITPLSFIGFILSSILMSPREQQESRVYNTCQNKYRFYYPIDDNFRHEISFQEFKNKLLEPGLVDRIVVTNKSVAKVYVRSSAPSPDQIGDDTVQVPVTGRNDRRNTSQYKYYFNIGSVESFEEKLEEAQEALRIDPHNYVPVTYVDELNWFQEVMRFGPTVLLLAVLYFMGRRVQGGMGVGGPGGKGGRGIFNIGKAHFTKMDKNAKNKVFFKDVAGCDEAKQEIMEFVHFLKNPKKYEQLGAKIPKGALLVGPPGTGKTLLAKATAGESGVPFLSISGSDFMEMFVGVGPARVRSLFQEARQCAPSIIFIDEIDAIGRARGRGGFSGGHDERESTLNQLLVEMDGFATTSGVVILAGTNRPDILDKALLRPGRFDRQITIDKPDIKGRDQIFLIYLNKLKLDHEASFYSQRLAALTPGFAGADIANVCNEAALIAARNESTIITMQHFEAAIDRVIGGLEKKNKVISKLERRTVAYHESGHAVAGWFLEHAEPLLKVTIVPRGTAALGFAQYVPNENLLMTKEQLFDVTCMTLGGRAAEQVLIGKISTGAQNDLEKVTKMTYAQVAVYGFSDKVGLLSFPQRDDGFEMSKPYSSKTAAIIDNEVREWVSKAYGRTVQLIEEHKEHVAQIAELLLEKEVLHQEDLVRVLGERPFKSHEPTNYDIFKQGFEEENKETKDNPENKTVEDNGSPPVVPEVVPL

>Sme2.5_05222.1_g00004.1

MAENEGSSSSTSRGQLLRPTITLPPRNSMESLFSGGSSGISPGPMTLVSSFFSDNDPDSECRSFSQLLAGAVTPPAGFPGARPGFPPLTPPGTAAISQLPTPAFAVPPGLSPTSLFDGFFSPGQGPFGMSHQQVLTQFTAQASQAQSQMQIQPNYSSSATAVAPSMSQFQSLTSNAAANKQIPPTLDPNIAKESSDVSLSDQRSEPACFAVDKPADDGYNWRKYGQKQVKGSEYPRSYYKCTHPNCPVKKKVERSLDGQVTEIIYKGQHNHQPPQASKRSKESGNPNGSYNPYELSSEGLTGNFNKSKEGEPSYSLRMKDQESSQANDQTSGSSDSEEVGNAETRVDGSYIDERESKRRAVEVQSSEAACSHRTVAEPRIIVQTTSEVDLLDDGYRWRKYGQKVVKGNPYPRSYYKCTSQGCNVRKHVERAASDPKAVITTYEGKHNHDVPAARNSSHNTANNAVSQLRPNFPVVDKPAAMRRADFQSNEQQPIALLRFKEEQIT

>Sme2.5_06310.1_g00004.1

MILSVDMMKSFFNATQMLESQVEELLQELKPNCLISDMCFPWTTNVAKRINIPRIVFHGMGSFSLLCLHSLRDGKLLESITCDTEYFSVPGLPDKVEVTKAQLKALVDPCNAEWREFGDQMKEAEDKAYGIVVNSFEELEPQYVQALKKAKGKKVWTVGPVSLCNKDTQDKVERGNTASIDEHYCLKWLDSRKPDSVLFVCLGSLSRLPTSQMIELALGLESSKRPFLWVVRNISDEFAKWLNEENFNERVEKQGILIQGISVGVPMITWPLFAEQFCNEKLIINVLNTGVKGGMENPVVFLDEEKVGTKVKKDKIKMVIERVMGEEEEAQMRRNRAKMFGEMAIMAMEEGDHVAQEVKRLSDENKLREMLSVMCDNYNSLRKQYMSNCSASGMSRKRKAENSRLFDARFHGINSELSCNKSRLVHHDHIQLKNINSTHYIRTTQASHDTSLIVKDGYEWRKYGQKVTRDNPYPRAYFKCSFAPTCPVKKKVQRSADDKSIIVATYEGEHNHSKVLLETGEGSSDQLVNGNRTISINTAAIRRSHNKQPEFHKLLIDQMASSLIKDPKFDAALAAALSRKTILPHK

>Sme2.5_06988.1_g00004.1

MSSSPCRTEFLTIPPGISPAALLDSPFMLPSSMIPTENGTQILNCIESRKNEIPQQEEEFIKKRRGIFSAGISPNNSSDDGYTWRKYGQKHVKGSNYPRSYYKCTQQNCPVRKKVECAPSGQVTEIVYINGAHNHPKTQNLRRKTTDDSYVVSQENGSSIWRNDQKFEHNEDIAGCNELERLSSASVLSDVSDPMLSNNLKSNESNGTHELSPTLTIFDGEDEDLATQEGYFLGDGIDEYELESKRRKKDDCLAEPSLLSRTVREPKVVLQVASEIDILEDGYRWRKYGQKVVKGNPNPRSYYKCTSAGCLVRKHVERASDDLKSVITTYEGKHNHEVPSANKTNGVSGNVRSASMLNNGQQTCTTTSRKSLKDSNIRVQFQDLPIPFERKHFMGSEYLRPSFGASYLGDLSFGASSLQLPDFPLPLPLPSRLSFPARQNEPRLGDFHLNNHFLLPNGASNPFLADGNTQHINNDSNNSRLLKAKDETQYWT

>Sme2.5_07339.1_g00001.1

MEKVRDLDKKKLMNELTQGKEFVKQLKRQIGPLASPQECDLLIGKILCTLEKSLSILNLKALLLEGGINNPNNSTSSSSSISFPGNNSNNSPKREVLDSSRDQLSKSMVSKKRKKSQQWTNQTSIFVNGVEGPQEDGYSWRKYGQKDILGANHPRAYYRCTHRNTQGCLATKQVQKSDGNSTMFEVTYKGRHSCKAAQSEIFSLGNQKRHKHKTKQEQNMLIFNSEPNHKVENFNITTKEEAFTPFPFSPTPLQLEETKFFSDSIAPFSSPIAPELSPYLSMLTSQNEEFGMMLQSSDSDLTELISTPTSISNSSFGGDCNLSEDFEPNLTFDIEEFLQLI

>Sme2.5_08092.1_g00001.1

MDAVFRKSIRGGVVLKEDTKIKADHEEDDINCLKVGKGRKSVHHEDDNSKSSQQKDLEEDDQLESAKADMEVVKEENQRLKKHLDRIMKDYRNLQMQFQEVAQKTNGVKHDEAELVSLSLGRTSSDTKKELYKLILSKNENEEEEDNLALGLDCNFQWHAITPSKSSPSNLSPENSSGEVKDDEKGAETWPPQKVLKTMRNEEDDVAQQNPTKRAKVSVRVRCDTPTMNDGCQWRKYGQKIAKGNPCPRAYYRCTVAPSCPVRKQVQRCIQDMSILITTYEGTHNHPLPQSATSMAFTTSAAASMLLSGSSSSGSGPTSTTGSATSTALNHYLSYNSKPNPFYLPNSSISSSSHSQYPTITLDLTSGSSISSLPVHNHRMSSNYPPRYNNSSTNILNFSSYDSNMSWSNGNFNQASQSYLQSNIISAMQPTQTNLSPQDTITAATKAIASDPKFQSALAVALTSIIGSRGANHHIDEKSEQNLKVNQPFPVLCSLPSTSHGDYKD

>Sme2.5_11773.1_g00001.1 /partial

RCNRMGLEVILNWEIALTRKRDVGSLTPHVHVMYRRHGQVESSGVMLCNDHRLGRDNLFTDEEISSAFPVIAHLVLLMLVKDIDMENSSSDLNRAIAGLIRGREFTQQLKEIIKKPRGEVANVMAEGLVAKIMDSFSETLSVINNSDEAKSPEDYSSASCKSSDRRGCYKRRKTSESNIKETSDLVDDGHAWRKYGQKQILHSTYPRHYFRCTHKYDQKCQASKQVQKIQDNPQLFRTTYYGHHTCKAFPRVSQIILDSPLDGNPNYISFDQNPTTFPSIKQEEVFSFYPKIEDQIQSSSSDYLLPNDHDHLTFEASCGHMQSPDVMSSGVSTTTTTSNDNLEIGIDFEECLWNFEGCS

>Sme2.5_14019.1_g00001.1 /partial

MDQNNNQQLYFHPIQPTQFSQQNFLAASPTTVTCVPTTTTTSPEWIDIQQQQHMMHDNVDPDLTLPMKTPLIKTYKGKNQPVRVFYEVLLEELTNDKWAWHKFGQKLLKGFPFPRNYYKCSAKLCEAKKTIEKSPTNENYFLVSYSGEHSHDPPTYRRPLIFYNSSSKYKLPRGINIVPKALSVNTSSVSSSKHAKRSKIDVSSICPIATTLKIESNNEMVNAVVENKNDVEEEENVNEDI

>Sme2.5_14251.1_g00004.1 /partial

AASPTTVTCVPTTTTTSPEWIDIQQQQHMMHDNVDPDLTLPMKTPLIKTYKGKNQPVRVFYEVSLEELTNDKWAWDKFGQKLLKGFPFPRNYYKCSAKLCKAKKTIEKSPTNENYFLVSYSGEHGHDPPTYPRPLIFYNSSSKYKLPRGINIVPKALSVNTSSVSSSKRAKCSKIDVFSICPTATTLKIESNNEMVNAVVENKNDVEEEENVNEDIIMEFEEHQGVTSST

>Sme2.5_15021.1_g00001.1

MEEIEEANRVAVESCHRVISMLSQPHDQEQFGNLVKETGEAVHKFKKVVTLLNSNLGHARVRKAKKILTPFPHNLLLETPSCKIDDQPKPLHLLPNTRLEIGSNVNRTHPSLELSSHSKNPLQLAQQTPLSSYHFVQQQQQQQLQQQQQRRYQLQQQQMKQQADMMYRRSNSGISLNFDSSTCTPTMSSTRSFISSLSIDASVANMDGNAFHFIGPSRSADESSFQHKKRCSGRGEEGSVKCGSSGIALRIRAHFNTRNGAPEEERREVKHRVKRSIKVPAISNKLADIPPDEYSWRKYGQKPIKGSPHPRGYYKCSSMRGCPARKHVERCLEEPSMLIVTYEGEHNHPRLPSQSANA

>Sme2.5_17732.1_g00001.1

MNAENQRLRGMLTQVSNNYTALQMHLASLMQRQQQVSTTSNTHDHKIVEAKSDQEKIVPSGDELSHNSHSLSEERTVSASPRNNIEVTRHTIGREESPESESWIAIKVPKLISSMPNGQTTEATMRKARVSVRARSEAPLISDGCQWRKYGQKVAKGNPCPRAYYRCTMAVGCPVRKQVQRCAEDGAILNTTYEGRHNHPLPSAAMAMASTTSAAANMLLSGSMPSVDGALMNPNFPARTMLPCSSSMATISASAPFPTITLDLTQNQNALPYNQTSAQSQVPFQSGPQHTNFITSMPPPPIPHNQSKFSGLEVSQPQQLQHVKNHPLFTQTLTAAITADPNFTAAVAAAISSIITSPGKQQ

>Sme2.5_18444.1_g00001.1 /partial

CNRFLMQDSYYSTFARSPQRSQSAVCLSLRVRDAGALICRADTAFTPHSQMSYPHCLSSPSIIMLSYLSFGLTHLGRLGECHHTWISVRDSQQNFLAASPTTVTCVVTTTITPPEWIDIQQQQHMMHDNVDPYLTLPVKTPLIKTNKGKNQPIRVFYEVLLEELTNDKWAWHKFGQKLLKGFPFPRNYYKCSAKLCKAKKTIEKSPTNENYFLVSYSGERSHDPPIYPRPLIFYNSSSKYKLPRGINIVPKALSVNTSSVSSSKRAKRSKIDVSSICPTTTTLKIESNNEMVKAVVDENVKRN
