# Supplementary material for: Identification of WRKY gene family and characterization of cold stress-responsive WRKY genes in eggplant
Source: PeerJ. 2020 Mar 17;8:e8777. doi: 10.7717/peerj.8777 (PMC7083166; doi:10.7717/peerj.8777)
Supplement: Data S6 [file peerj-08-8777-s006.docx]

**Data S6: The misannotated WRKY proteins predicted in eggplant genome**

| Gene ID | Misannotated type |
| --- | --- |

| Sme2.5_00009.1_g00044.1 | No typical WRKY domain |
| --- | --- |
| Sme2.5_00013.1_g00034.1 | No typical WRKY domain |
| Sme2.5_00378.1_g00006.1 | Incomplete WRKY domain |
| Sme2.5_00708.1_g00006.1 | No typical WRKY domain |
| Sme2.5_01077.1_g00011.1 | No typical WRKY domain |
| Sme2.5_01363.1_g00005.1 | Incomplete WRKY domain |
| Sme2.5_01363.1_g00007.1 | No typical WRKY domain |
| Sme2.5_01461.1_g00006.1 | Incomplete WRKY domain |
| Sme2.5_02156.1_g00007.1 | No typical WRKY domain |
| Sme2.5_02226.1_g00005.1 | No typical WRKY domain |
| Sme2.5_03023.1_g00004.1 | No typical WRKY domain |
| Sme2.5_03997.1_g00008.1 | No typical WRKY domain |
| Sme2.5_06423.1_g00002.1 | Incomplete WRKY domain |
| Sme2.5_06927.1_g00005.1 | No typical WRKY domain |
| Sme2.5_09317.1_g00001.1 | No typical WRKY domain |
| Sme2.5_11716.1_g00001.1 | No typical WRKY domain |
| Sme2.5_14251.1_g00001.1 | No typical WRKY domain |
| Sme2.5_14251.1_g00003.1 | No typical WRKY domain |
| Sme2.5_15262.1_g00001.1 | No typical WRKY domain |
